# Review on Vehicle Detection Technology for Unmanned Ground Vehicles

**DOI:** 10.3390/s21041354

**Published:** 2021-02-14

**Authors:** Qi Liu, Zirui Li, Shihua Yuan, Yuzheng Zhu, Xueyuan Li

**Affiliations:** 1 School of Mechanical Engineering, Beijing Institute of Technology, Beijing 100089, China; 3120195257@bit.edu.cn (Q.L.); 3120195255@bit.edu.cn (Z.L.); 3120205197@bit.edu.cn (Y.Z.); lixueyuan@bit.edu.cn (X.L.); 2 Department of Transport and Planning, Faculty of Civil Engineering and Geosciences, Delft University of Technology, Stevinweg 1, 2628 CN Delft, The Netherlands

**Keywords:** unmanned ground vehicles, sensor, vehicle detection, simulation platform, dataset

## Abstract

Unmanned ground vehicles (UGVs) have great potential in the application of both civilian and military fields, and have become the focus of research in many countries. Environmental perception technology is the foundation of UGVs, which is of great significance to achieve a safer and more efficient performance. This article firstly introduces commonly used sensors for vehicle detection, lists their application scenarios and compares the strengths and weakness of different sensors. Secondly, related works about one of the most important aspects of environmental perception technology—vehicle detection—are reviewed and compared in detail in terms of different sensors. Thirdly, several simulation platforms related to UGVs are presented for facilitating simulation testing of vehicle detection algorithms. In addition, some datasets about UGVs are summarized to achieve the verification of vehicle detection algorithms in practical application. Finally, promising research topics in the future study of vehicle detection technology for UGVs are discussed in detail.

## 1. Introduction

The unmanned ground vehicle (UGV) is a comprehensive intelligent system that integrates environmental perception, location, navigation, path planning, decision-making and motion control [1]. It combines high technologies including computer science, data fusion, machine vision, deep learning, etc., to satisfy actual needs to achieve predetermined goals [2].

In the field of civil application, UGVs are mainly embodied in autonomous driving. High intelligent driver models can completely or partially replace the driver’s active control [3,4,5]. Moreover, UGVs with sensors can easily act as “probe vehicles” and perform traffic sensing to achieve better information sharing with other agents in intelligent transport systems [6]. Thus, it has great potential in reducing traffic accidents and alleviating traffic congestion. In the field of military application, it is competent in tasks such as acquiring intelligence, monitoring and reconnaissance, transportation and logistics, demining and placement of improvised explosive devices, providing fire support, communication transfer, and medical transfer on the battlefield [7], which can effectively assist troops in combat operations.

**Figure 1:**
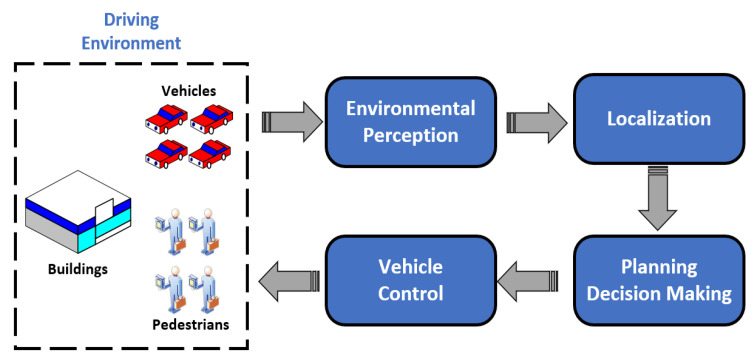
Technical framework for UGVs.

The overall technical framework for UGVs is shown in Figure 1. It is obvious that environmental perception is an extremely important technology for UGVs, including the perception of the external environment and the state estimation of the vehicle itself. An environmental perception system with high-precision is the basis for UGVs to drive safely and perform their duties efficiently. Environmental perception for UGVs requires various sensors such as Lidar, monocular camera and millimeter-wave radar to collect environmental information as input for planning, decision making and motion controlling system.

Environment perception technology includes simultaneous localization and mapping (SLAM), semantic segmentation, vehicle detection, pedestrian detection, road detection and many other aspects. Among various technologies, as vehicles are the most numerous and diverse targets in the driving environment, how to correctly identify vehicles has become a research hotspot for UGVs [8]. In the civil field, the correct detection of road vehicles can reduce traffic accidents, build a more complete ADAS [9,10] and achieve better integration with driver model [11,12], while in the field of military, the correct detection of military vehicle targets is of great significance to the battlefield reconnaissance, threat assessment and accurate attack in modern warfare [13].

**Figure 2:**
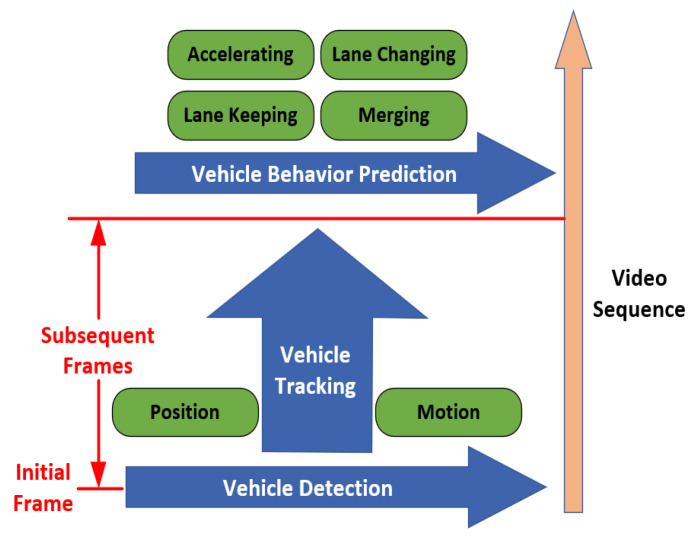
The overall framework of vehicle recognition technology for unmanned ground vehicles (UGVs).

The complete framework of vehicle recognition in UGVs autonomous driving system is portrayed in Figure 2. Generally, vehicle detection is used to extract vehicle targets in a single frame of an image, vehicle tracking aims to reidentify positions of the vehicles in subsequent frames, vehicle behavior prediction refers to characterizing vehicles’ behavior basing on detection and tracking in order to make a better decision for ego vehicle [14]. For tracking technology, readers can refer to [15,16], while for vehicle behavior prediction, [17] presented a brief review on deep-learning-based methods. This review paper focuses on the vehicle detection component among the complete vehicle recognition process, summarizes and discusses related research on vehicle detection technology with sensors as the main line.

**Figure 3:**
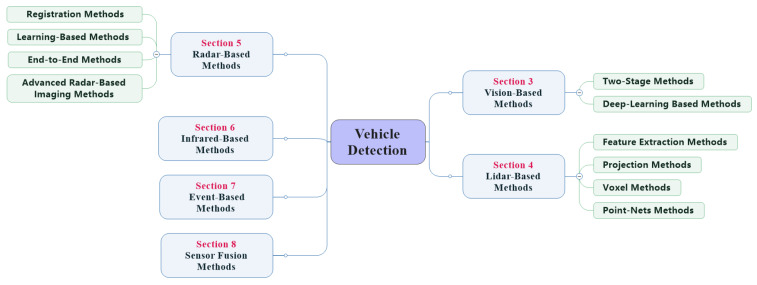
Structure of vehicle detection algorithm overview in this survey.

This article is organized as followed. Section 2 introduces the commonly used sensors on UGVs and compares the pros and cons of different sensors under different application scenarios. Section 3, Section 4, Section 5, Section 6, Section 7 and Section 8 systematically summarizes and compares the research works related to vehicle detection using different sensors, the structure of the vehicle detection overview is illustrated in Figure 3. Section 9 introduces the simulation platform related to UGVs, which is convenient for simulation tests of the vehicle detection algorithm. Section 10 introduces the datasets to verify the actual effect of the vehicle detection algorithm. Section 11 summarizes and looks forward to the research focus and direction of vehicle detection technology.

## 2. Sensors for Vehicle Detection

The operation of UGVs requires a persistent collection of environmental information, and the efficient collection of environmental information relies on high-precision and high-reliability sensors. Therefore, sensors are crucial for the efficient work of UGVs. They can be divided into two categories: Exteroceptive Sensors (ESs) and Proprioceptive Sensors (PSs) according to the source of collected information.

ESs are mainly used to collect external environmental information, specifically vehicle detection, pedestrian detection, road detection, semantic segmentation, commonly used ESs include Lidar, millimeter-wave radar, cameras, ultrasonic. PSs are mainly used to collect real-time information about the platform itself, such as vehicle speed, acceleration, attitude angle, wheel speed, and position, to ensure real-time state estimation of UGV itself, common PSs include GNSS, and IMU.

Readers can refer to [18] for detailed information on different sensors. This section mainly introduces ESs that have the potential for vehicle detection. ESs can be further divided into two types: active sensors and passive sensors. The active sensors discussed in this section include Lidar, radar, and ultrasonic, while passive sensors include monocular cameras, stereo cameras, omni-direction cameras, event cameras and infrared cameras. Readers can refer to Table 1 for the comparison of different sensors.

### 2.1. Lidar

Lidar can obtain object position, orientation, and velocity information by transmitting and receiving laser beam and calculating time difference. The collected data type is a series of 3D point information called a point cloud, specifically the coordinates relative to the center of the Lidar coordinate system and echo intensity. Lidar can realize omni-directional detection, and can be divided into single line Lidar and multi-line Lidar according to the number of laser beams, the single line Lidar can only obtain two-dimensional information of the target, while the multi-line Lidar can obtain three-dimensional information.

Lidar is mainly used in SLAM [19], point cloud matching and localization [20], object detection, trajectory prediction and tracking [21]. Lidar has a long detection distance and a wide field of view, it has high data acquisition accuracy and can obtain target depth information, and it is not affected by light conditions. However, the size of Lidar is large with extremely expensive, it cannot collect the color and texture information of the target, the angular resolution is low, and the long-distance point cloud is sparsely distributed, which is easy to cause misdetection and missed detection, and it is easily affected by sediments in the environment (rain, snow, fog, sandstorms, etc.) [22], at the same time, Lidar is an active sensor, and the position of the sensor can be detected by the laser emitted by itself in the military field, and its concealment is poor.

### 2.2. Radar

Radar is widely used in the military and civilian fields with important strategic significance. The working principle of a radar sensor is like that of Lidar, but the emitted signal source is radio waves, which can detect the position and distance of the target.

Radars can be classified according to the different transmission bands, and the radars used by UGVs are mostly millimeter-wave radars, which are mainly used for object detection and tracking, blind-spot detection, lane change assistance, collision warning and other ADAS-related functions [18]. Millimeter-wave radars equipped on UGVs can be further divided into “FMCW radar 24-GHz” and “FMCW radar 77-GHz” according to their frequency range. Compared with long-range radar, “FMCW radar 77-GHz” has a shorter range but relatively high accuracy with very low cost, therefore almost every new car is equipped with one or several “FMCW radar 77-GHz” for its high cost- performance. More detailed information about radar data processing can refer to [23].

Compared with Lidar, radar has a longer detection range, smaller size, lower price, and is not easily affected by light and weather conditions. However, radar cannot collect information such as color and texture, the data acquisition accuracy is general, and there are many noise data, the filtering algorithm is often needed for preprocessing, at the same time, radar is an active sensor, which has poor concealment and is easy to interfere with other equipment [24].

### 2.3. Ultrasonic

Ultrasonic detects objects by emitting sound waves and is mainly used in the field of ships. In terms of UGVs, ultrasonic is mainly used for the detection of close targets [25], ADAS related functions such as automatic parking [26] and collision warning [27].

Ultrasonic is small in size, low in cost, and not affected by weather and light conditions, but its detection distance is short, the accuracy is low, it is prone to noise, and it is also easy to interfere with other equipment [28].

### 2.4. Monocular Camera

Monocular cameras store environmental information in the form of pixels by converting optical signals into electrical signals. The image collected by the monocular camera is basically the same as the environment perceived by the human eye. The monocular camera is one of the most popular sensors in UGV fields, which is strongly capable of many kinds of tasks for environmental perception.

Monocular cameras are mainly used in semantic segmentation [29], vehicle detection [30,31], pedestrian detection [32], road detection [33], traffic signal detection [34], traffic sign detection [35], etc. Compared with Lidar, radar, and ultrasonic, the most prominent advantage of monocular cameras is that they can generate high-resolution images containing environmental color and texture information, and as a passive sensor, it has good concealment. Moreover, the size of the monocular camera is small with low cost. Nevertheless, the monocular camera cannot obtain depth information, it is highly susceptible to illumination conditions and weather conditions, for the high-resolution images collected, longer calculation time is required for data processing, which challenges the real-time performance of the algorithm.

### 2.5. Stereo Camera

The working principle of the stereo camera and the monocular camera is the same, compared with the monocular camera, the stereo camera is equipped with an additional lens at a symmetrical position, and the depth information and movement of the environment can be obtained by taking two pictures at the same time through multiple viewing angles information. In addition, a stereo vision system can also be formed by installing two or more monocular cameras at different positions on the UGVs, but this will bring greater difficulties to camera calibration.

In the field of UGVs, stereo cameras are mainly used for SLAM [36], vehicle detection [37], road detection [38], traffic sign detection [39], ADAS [40], etc. Compared with Lidar, stereo cameras can collect more dense point cloud information [41], compared with monocular cameras, binocular cameras can obtain additional target depth information. However, it is also susceptible to weather and illumination conditions, in addition, the field of view is narrow, and additional calculation is required to process depth information [41].

### 2.6. Omni-Direction Camera

Compared with a monocular camera, an omni-direction camera has too large a view to collect a circular panoramic image centered on the camera. With the improvement of the hardware level, they are gradually applied in the field of UGVs. Current research work mainly includes integrated navigation combined with SLAM [42] and semantic segmentation [43].

The advantages of omni-direction camera are mainly reflected in its omni-directional detection field of view and its ability to collect color and texture information, however, the computational cost is high due to the increased collection of image point clouds.

### 2.7. Event Camera

An overview of event camera technology can be found in [44]. Compared with traditional cameras that capture images at a fixed frame rate, the working principle of event cameras is quite different. The event camera outputs a series of asynchronous signals by measuring the brightness change of each pixel in the image at the microsecond level. The signal data include position information, encoding time and brightness changes.

Event cameras have great application potential in high dynamic application scenarios for UGVs, such as SLAM [45], state estimation [46] and target tracking [47]. The advantages of the event camera are its high dynamic measurement range, sparse spatio-temporal data flow, short information transmission and processing time [48], but its image pixel size is small and the image resolution is low.

### 2.8. Infrared Camera

Infrared cameras collect environmental information by receiving signals of infrared radiation from objects. Infrared cameras can better complement traditional cameras, and are usually used in environments with peak illumination, such as vehicles driving out of a tunnel and facing the sun, or detection of hot bodies (mostly used in nighttime) [18]. Infrared cameras can be divided into infrared cameras that work in the near-infrared (NIR) area (emit infrared sources to increase the brightness of objects to achieve detection) and far-infrared cameras that work in the far-infrared area (to achieve detection based on the infrared characteristics of the object). Among them, the near-infrared camera is sensitive to the wavelength of 0.15–1.4 μm, while the far-infrared camera is sensitive to the wavelength of 6–15 μm. In practical applications, the corresponding infrared camera needs to be selected according to the wavelength of different detection targets.

In the field of UGVs, infrared cameras are mainly used for pedestrian detection at night [49,50] and vehicle detection [51]. The most prominent advantage of an infrared camera is its good performance at night, Moreover, it is small in size, low in cost, and not easily affected by illumination conditions. However, the images collected do not contain color, texture and depth information, and the resolution is relatively low.

## 3. Vehicle Detection: Vision-Based Methods

Vision-based vehicle detection can be divided into two-stage methods and one-stage methods according to the inspection process. These two methods will be discussed in detail in the following content.

### 3.1. Two-Stage Methods

**Figure 4:**
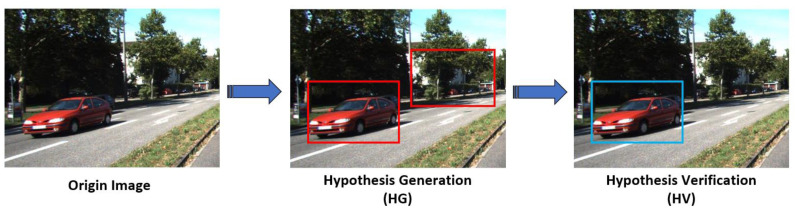
Detection flowchart of two-stage methods. Schematic is taken from KITTI dataset [52].

Vision-based two-stage vehicle detection method usually follows two steps: hypothetical generation (HG) and hypothetical verification (HV). The purpose of the HG step is to generate a candidate region that may contain vehicles in the captured image, represents the region of interests (ROIs), while the HV step aims to identify the presence of a vehicle in ROIs. The detection process of two-stage methods is described in Figure 4.

#### 3.1.1. Hypothetical Generation (HG)

Various HG methods with vision sensors can be divided into three categories: appearance-based methods, motion-based methods and stereo-based methods. Moreover, related works of appearance-based and motion-based methods are summarized in Table 2 related works of stereo-based methods are summarized in Table 3.

- Appearance-based Methods

The appearance-based method depends on the prior knowledge of the vehicle to generate the ROIs in an image. Some important cues to extract vehicle features including color, edges, corners, symmetry, texture, shadow and vehicle lights are reviewed in the following content.

**(a)**  **Color** 

Color provides rich information in an image, ensuring the great potential for scene understanding. In general, colors of vehicle body and lights are evenly distributed and have large discrimination from the road surface and image background, thus color information can be extracted to segment vehicles from background to generate ROIs.

In [53], a conventional RGB color space was used to generate ROIs. Firstly, all red areas in the image were extracted through the RGB color space, then prior knowledge that the vehicle brake lights have the same shape, size and symmetrical distribution was used to design a similar scale calculation model to extract the position of brake lights to be the final ROIs.

Since the RGB color space is sensitive to changes of illumination, in [54], ROIs were also generated by detecting brake lights, but the color space proposed is L*a*b, which is insensitive to the changes of illumination, Moreover, in [55], HSV color space was put forward to generate ROIs. In [56], RGB color space was combined with the background modeling method to reduce the impact of illumination, and the accuracy of the extracted ROIs was about 95%, but the real-time performance of the algorithm was therefore affected.

To achieve a better balance between accuracy and real-time performance, in [57], ROIs were extracted based on HSV color space with the “convex hull” operation to filter out noisy points in the image, then the boundary of ROIs was fitted to make it smooth, the accuracy of the algorithm was about 91.5% with running time about 76 ms/fps. In [58], ROIs were first extracted using HSV color space, and then RGB color space was utilized to further detect the vehicle lights in the ROIs to achieve the detection of emergency vehicles (vehicles with double flashes in an accident, ambulances, etc.). This work expanded the application scenarios and made a contribution to the early warning of vehicle behavior.

**(b)**  **Edges** 

Different views of the vehicle (especially front and rear view) contain different types of edge features, for instance, the horizontal edge features of the bumper and the horizontal and vertical edge features of the windows show a strong ability to generate ROIs.

In general, the changes of gray value on sides of edges in the image is faster than in other areas. Thus, a possible solution is to calculate the sum of the gray value of each row and column in the image to form an “Edge Map”, and preliminarily judge the location of edges in the image (potential location of ROIs) according to the peak gray value [59,60].

In addition to the implementation of “Edge Map” methods, the Sobel operator is another choice to extract vehicle edge features. In [61], Sobel operator was used to extract the left and right edge features of the vehicle, then the grayscale of the image was analyzed to extract the shadow of the vehicle, both were finally fused with a designed filter to generate ROIs, the accuracy of this approach is about 70%.

Moreover, several approaches were carried out to optimize Sobel operator in edge extraction. In [62], the “Scharr-Sobel” operator was established to highlight image edge features and reduce the complexity of the algorithm with an accuracy of 82.2%. In [63], the Sobel operator was combined with Hough transform to extract vehicle edge features, and a Faster-RCNN was trained for verification. In [64], the Sobel operator and Perwitt operator were combined to extract the edge features of vehicles for detection. The accuracy is different under different traffic conditions, which fluctuated between 70% and 90%.

**(c)**  **Corners** 

From the perspective of vehicle design, the shape of the vehicle can be generally regarded as a rectangle. The four corners that can form a rectangle in all corners detected in the image can be used as the basis for generating ROIs.

In [65], Harris corner detection model was proposed to extract corners in the image, and “corner mask” was designed to remove false-detected corners, then corners and color features were fused to generate ROIs, the detection accuracy of vehicles of different colors varied from 79.66% to 91.73%. In [66], a grayscale map was created to select appropriate threshold value to detect corners in the image, then, coordinate of all corners were calculated and paired between each other, and finally “Convex Hull algorithm” were carried out to generate ROIs.

**(d)**  **Symmetry** 

The front view and the rear view of the vehicle have obvious symmetry with respect to the vertical centerline, thus the location of the vehicle is able to be evaluated by detecting the area with high symmetry characteristics in the image to generate ROIs. The symmetry detection methods need to calculate the symmetry measurement in the image to generate a symmetry axis or center point for the vehicle by adapting the image pixel characteristics (grayscale, color, feature point, etc.).

The detection of symmetry features usually requires the extraction of edge features in the image. In [67], the edge features of the image were firstly extracted and 15 horizontal scan lines were generated to select the candidate areas, then, a symmetry measurement function was designed basing on contour features to extract the vehicle symmetry axis, and the k-means clustering was used to extract the central point and generate ROIs. The detection accuracy was about 94.6% with a running time of 80 ms/fps. In [68], the symmetry measurement function was also based on the contour feature to extract the symmetry axis and the center point, compared with [67], the author transplanted the program package to the Android system, and the detection accuracy was about 92.38%, with a running time of 33 ms/fps. Limited by the computing performance of the Android system, although this algorithm meets the requirements of good detection accuracy and real-time performance, it can only be used for the detection of simple scenes, and the results did not have a strong reference. The Canny operator was used in [69] to extract edge features, and combined with the two-frame difference method, the extracted edge was “amplified” to enhance its feature strength, so as to improve the accuracy of symmetry axis extraction. This method had been tested to achieve a better performance for dynamic vehicle detection.

Apart from extract image edge features first, a linear regression model was also used to extract vehicle symmetry axis in [70], Haar features with Adaboost classifier were trained for verification using an active learning method, with an accuracy of about 87% and running time of 40 ms/fps.

**(e)**  **Texture** 

The presence of the vehicle will cause the local intensity change in an image, and the rules of intensity change follow a certain texture form. Thus, the difference between the vehicle texture and the background environment can be used to extract ROIs from the image.

There were few studies on the algorithm of vehicle detection by extracting texture features. The main approaches include entropy [71], gray level co-occurrence matrix [72] and LBP [73]. In [71], the entropy value of each pixel was calculated in the image, and the area with high entropy value was regarded as the ROIs of possible vehicles. In [72], ROIs were extracted by calculating the gray co-occurrence matrix of images, compared with the simple calculation of image entropy, this method was a second-order statistic of pixels, with higher accuracy but larger computing cost. In [73], the texture feature of the image background (mainly referred to road area) was extracted by LBP method, and the location of the shadow was extracted by using the characteristic that the bottom shadow is similar to the texture feature of the road area, both were fused to generate ROIs. The texture features extracted by the LBP method were suitable for further classification by SVM.

**(f)**  **Shadow** 

The shadow area underneath the vehicle in the image is darker than the area of the road surface. The feature of brightness difference can be used to extract ROIs by investigating image intensity.

The conventional shadow-based method is to select an appropriate threshold (lower bound of road intensity) to segment the shadow areas. In [74], road areas in the image were first extracted, and the shadow areas were defined according to the intensity that is less than the threshold “m-3σ” to generate ROIs, where m and σ are the average and standard deviation of the road pixel frequency distribution, after which ROIs were verified by SVM. The detection accuracy is about 92% with a running time of 76 ms/fps.

Since the intensity of the shadow area is sensitive to changes of illumination, selecting a fixed threshold to segment the shadow area cannot be applied to various scenes. Therefore, an adaptive threshold algorithm is carried out in [75], the pixel ratio of each point in the image was first calculated, then two parameters α and β between 0 and 1 were selected and the areas whose pixel ratio between α and β were defined as shadows. Although this method solved the limitation of the fixed threshold methods, the selection of parameters α and β requires constant iteration, which makes it difficult to obtain an optimal solution. Thus, in [76], a “three threshold” method was presented based on the RGB color space combined with the ViBe algorithm to extract the shadow areas in order to further improve the robustness.

Different from the aforementioned literature by determining the threshold value as the lower bound of the intensity of the road area in the image to segment the shadow area, in [77], the rough upper bound of the intensity of the undercarriage was determined based on the “binary mask function” constrained by saturation and intensity difference Value to reduce false detection rate.

**(g)**  **Vehicle Lights** 

For nighttime vehicle detection, the performance of vision cameras is greatly affected due to the poorly illuminated conditions, therefore most cues summarized above are not reliable during nighttime detection. A salient feature of the vehicle is its headlights and taillights that can be extracted to represent ROIs of the vehicle.

One possible solution is to extract the features of vehicle lights from the image background by setting a specific threshold [78]. In [78], the threshold was selected as 30% of the maximum gray value in the threshold image to extract the position of the lights to generate ROIs. Since the gray value of the vehicle lights is various at different distances from the camera, and the roadside lighting equipment will also affect the environmental brightness, only setting a single threshold for detection is prone to false detection.

In order to solve this problem, in [79], the lower bound of the threshold value of lights was first calculated based on the grayscale image, then, the OTSU method was proposed to obtain the optimized threshold, the similarity measurement was finally calculated to match the extracted vehicle lights with Kalman filter for noisy reduction to generate ROIs. In [80], the “CenSurE” method was put forward based on the Laplacian of Gaussian (LOG) to detect areas with sharp intensity changes in the image to extract the features of the lights, and paired by detecting lights on the same horizontal line, this approach did not depend on a specific threshold and achieved a faster calculation speed than LOG.

Apart from the threshold method, some researchers used machine learning methods to extract features of vehicle lights. In [81,82], original images were first converted into grayscale images, then an Adaboost classifier with Haar features was trained to get the position of the vehicle lights, and finally, the similarity measurement was calculated to pair the lights.

The width lights and brake lights of vehicles are red, therefore the red areas can be detected according to the color-based method discussed above. In [83], RGB color space was used to extract features of vehicle lights, and then closing operation (one of the morphological operations) was performed to eliminate holes in the feature map. ln [84] HSV color space was proposed to extract features of car light, after which Gaussian filter was used for filtering and noise reduction, and non-maximum suppression (NMS) method was implemented to eliminate the overlapping area.

**(h)**  **Multiple Features Fusion** 

Since using a single feature for vehicle hypothesis generation is limited in different application scenarios, ROIs can be generated by fusing multiple features to improve the robustness and reliability of the detection system, however, it will increase the complexity and calculation time of the system. There is no conventional method to select which features and which algorithm to use for fusion, some related works are listed in Table 4.

- Motion-based Methods

The motion-based methods generate ROIs by extracting the changes of the moving vehicle relative to the background in the image sequence. Compared with the appearance-based method, it is able to achieve a more direct detection process without prior knowledge of the vehicle. Nevertheless, for a single frame image or a low-speed moving and a stationary vehicle in the image sequence, the method will fail. Related methods include the frame difference method, background modeling method and optical flow method.

**(a)**  **Frame Difference** 

The frame difference methods first calculate the absolute value of the grayscale difference between adjacent frames of the image sequence and then select a threshold to distinguish the background and foreground in the image. If the absolute value satisfies the threshold condition, it can be judged as the ROIs of moving vehicles.

In [88], a conventional two-frame difference method was proposed for vehicle detection. Although the two-frame difference method achieved a low computing cost, it was pointed out that if the detected object had a relatively uniform grayscale, the overlapping part of the moving objects in the image will appear to be “blank” [89]. Therefore, the three-frame difference method was established to solve this problem in [89]. Later, some researchers made further improvements to the three-frame difference method to better solve the problem of “blank holes” in the image. The three-frame difference method was combined with the Gaussian model in [90], while it was combined with the image contrast enhancement algorithm and morphological filtering in [91]. In [92] a five-frame difference method was designed for vehicle detection in low-speed motion.

**(b)**  **Background Modeling** 

This approach establishes a background model through the video sequence. It generates a hypothesis of moving vehicles through pixel changes with the assumption that the background is stationary. The main challenge background modeling needs to solve is the establishment and update of the background model.

The typical background modeling method is the Gaussian Mixture Model (GMM) proposed by [93]. The main idea of the method is to assume that all data points in the image are generated by a finite Gaussian distribution with unknown parameters. Due to the slow initialization of GMM and the inability to distinguish between moving objects and shadows [94], an adaptive GMM method was designed to solve this problem in [94], and adaptive GMM was combined with a vehicle underneath shadow features to improve the computational efficiency and robustness.

Another typical algorithm is the codebook algorithm [95], which is characterized by high calculation accuracy. An improved codebook algorithm based on the conventional codebook algorithm was designed in [96] to improve its computational efficiency in complex environments.

In addition, the ViBE algorithm was proposed in [97]. This approach first selected a pixel and extracted the pixel value in the neighborhood of this pixel at a current and previous time to form a point set, then the pixel value of the selected pixel was compared with the pixel value in point set to determine whether the pixel belonged to the background, in general, the ViBE algorithm was able to achieve strong real-time performance, and overall has a relatively good background detection effect. Moreover, in [98], an adaptive ViBE algorithm was designed based on the ViBE algorithm to improve the background update efficiency for scenes with changing illumination.

Machine learning methods have also been applied to background modeling by researchers. In [99], the feature input was the first to fourth order statistics of the grayscale of the image, and the output was the appropriate morphological parameters to dynamically adjust the extracted background, the author tested it under the condition of sudden illumination changes and result showed better robustness.

**(c)**  **Optical Flow** 

The optical flow methods obtain the motion information of the object by matching the feature points between two adjacent frames in the image sequence or calculating the pixel changes, the return value is the optical flow vector of the object (describes the instantaneous velocity of a certain point in the image), and the optical flow at each point in the image constitutes an optical flow field to generate ROIs for moving objects.

Optical flow can be divided into dense optical flow and sparse optical flow. Dense optical flow is also called global optical flow, and it calculates the optical flow field of the whole image or a certain area, the registration n result is accurate but the computing cost is large. The typical methods are the Horn–Schunck (HS) optical flow method and its extension [100]. Sparse optical flow is also called local optical flow to calculate the optical flow field at some specific point, which improves the calculation efficiency but reduces the registration accuracy. The typical methods are the Lucas–Kanad (LK) optical flow method and its extension [101].

In [102], pyramid LK optical flow was proposed with the fusion of edge feature extraction, and k-means clustering was finally used to detect vehicles. In [103], the fusion of HS optical flow method and median filtering was proposed to achieve vehicle detection. In [104], ROIs were first extracted based on CNN, then, the Haar feature was utilized to extract feature points for ROIs, and finally, the LK optical flow method and k-means clustering were combined to achieve vehicle detection.

- Stereo-based Methods

It should be noted that the aforementioned appearance-based methods and motion-based methods can also be carried out in the images collected by stereo vision. Compared with monocular cameras, stereo cameras can obtain scene depth information, which enables more information for vehicle detection. Typical hypothesis generation methods using stereo camera include Inverse Perspective Mapping and disparity map.

**(a)**  **Inverse Perspective Mapping** 

The Inverse Perspective Mapping (IPM) refers to transforming the image collected by the stereo camera from the camera coordinate system to the world coordinate system through the rotation and translation transformation, and the result is a top view image without disparity. Depth information of roads and objects can be obtained through IPM, providing intuitive information for vehicle detection.

In [105,106], IPM was used to convert the left and right images from stereo camera into two top view images respectively; then, pixel difference between top view images was calculated, the areas with a non-zero difference were regarded as possible vehicles, and the locations of the ROIs were finally determined by the polar coordinate histogram. In [107], IPM was fused with a background modeling method to detect vehicles in motion. Moreover, IPM can also be used to obtain more information about the vehicle to be detected. In [110], ROIs were extracted based on IPM and the distance between the vehicle and the camera center was obtained, while in [111], IPM was combined with CNN to obtain the location, size and attitude angle of vehicles.

Using IPM for vehicle detection is easy to implement and computationally efficient, but this approach needs to be assumed that the road surface is completely flat and the road area in the image is large, so it is not suitable for vehicle detection in complex or unstructured scenes. Since the geometric information of the road can be extracted intuitively from the top view, IPM can often implement for road detection [128,129,130], some researchers used IPM to detect road areas to assist vehicle detection.

In [108], the drivable areas were first generated through IPM as preliminary determined ROIs, then vehicle lights were extracted to generate precise ROIs. In [109], road edges were extracted through IPM to first generate ROIs, the left and right cameras were then transformed to top view images and pixel differences were compared to achieve vehicle detection.

**(b)**  **Disparity Map** 

The difference between the corresponding pixels between left and right images is represented as disparity, calculating disparity of all the image points forms the disparity map, in addition, the disparity is negatively related to the distance between the image point and the camera. Planes in the image can be extracted by statistically analyzed the disparity distribution to generate areas contain an object with flat features (i.e., Side of the vehicle).

The derivation and calculation of the disparity map were reviewed in [131]. In order to make better use of the disparity map for object detection, some researchers have optimized the traditional disparity map to directly acquire scene information. For instance, the V-disparity map [112] that can be used to extract planes parallel to the camera horizontal plane (usually referred to as road areas), the UV-disparity map [113] that combined the U-disparity map on the basis of the V-disparity map to further extract planes perpendicular to the camera horizontal plane to realize 3D reconstruction of the environment.

In [114], a V-disparity map was combined with an optimized evolutionary algorithm (EA) for vehicle ROIs generation. In [115], the UV-disparity map and DCNN were combined to jointly extract vehicle ROIs. In [116], V-disparity maps with Hough transform were first utilized to extract road areas, and then a U-disparity map was used to generate ROIs, in addition, the distance of the vehicles was also derived based on depth information. In [118], a stereo camera was fused with millimeter-wave radar for vehicle detection, where stereo images were acquired to detect nearby vehicles through UV-disparity maps. In [119], ROIs were extracted based on a UV-disparity map and verified by Faster-RCNN.

Vehicle detection based on original disparity maps were also carried out by some researchers. One main approach is clustering point clouds data to extract vehicle information. In [120], a mean-shift algorithm was proposed based on a semi-dense disparity map to achieve vehicle detection and tracking. In [121], K-neighbor clustering with frame difference method and fast corner detection method was put forward to realize vehicle detection, while in [123], K-neighbor clustering was combined with optical flow, moreover, in [122], DBSCAN was used for ROIs generation. In [124], CNN was trained to generate semantic maps, then clustering based on the DFS method was performed to detect vehicles.

In addition, some researchers did not process the original disparity map for vehicle detection based on the clustering method. A typical method was designed in [117], the author first calculated the disparity map and combined the depth information to achieve 3D reconstruction, then, the RANSAC method was used to fit the road surface, and the areas above a certain threshold of the ground were regarded as ROIs, which were then matched with the predefined CAD wireframe model of the vehicle for verification.

**(c)**  **Optical Flow** 

The application of the optical flow method in stereo vision is similar to that in monocular vision. In general, feature points of interest are usually extracted through a single camera, and the three-dimensional coordinate of the object to be detected is determined by combining the disparity map and depth map.

In [126], the optical flow was used to detect vehicles moving in opposite directions, while the Haar feature was extracted to detect vehicles moving in the same direction. In [125], the 3D optical flow was obtained by matching feature points and the motion state of ego vehicle generated by the visual odometer, then 3D optical flow was projected to the aerial view to realize vehicle detection. In [127], optical flow and vehicle motion estimation model were designed based on stereo vision, then optical flow generated by camera motion (COF) was estimated by using the motion information of vehicle and the depth information of scene, and mixed optical flow (MOF) of scene was estimated by using HS algorithm. Finally, MOF and COF were differential calculated with elimination of static objects in the background, and vehicle detection was achieved by morphological filtering.

#### 3.1.2. Hypothetical Verification (HV)

The input of the HV stage is the set of hypothesis locations generated from the HG stage. During the HV stage, solutions are carried out to validate whether there is a true vehicle in ROIs. Various HV methods can be divided into two categories: template-based methods and classifier-based methods.

- Template-based Methods

The template-based methods need to establish the predefined vehicle feature template basing on different types of vehicle images, then the similarity measurement is put forward by calculating the correlation between the templates and ROIs.

Due to various types, shapes, and brands of vehicles, it is necessary to establish a generic template that can represent the common characteristics of the vehicle in order to make the template more widely used. Typical feature templates included as follows: a template that combines rear windows and license plates [132], a rectangular template with a fixed aspect ratio [133], and an “inverted U-shaped” template with one horizontal edge and two vertical edges [134].

In the same image, the vehicle could appear in different size and shape related to its distance and captured perspective from the camera [9], therefore, the traditional template matching method did not achieve good robustness, and the establishment of a dynamic template was significant to improve the efficiency of verification. In [135], a hybrid template library was established for matching, fusing four kinds of feature templates including vehicle wireframe model, texture model, image flatness and image color consistency. In [136] a deformable vehicle wireframe model was established with logistic regression to achieve vehicle detection, the model was composed of several discrete short line segments, and it could be dynamically adjusted to adapt to different capture perspectives and distance of vehicles in image, through the translation and rotation transformation of the short line segments.

A large number of modern vehicle datasets were collected in [137], vehicle appearances were analyzed through active learning methods, and a multi-view variable vehicle template library was then established. It should be noted that this approach was different from the conventional vehicle template mentioned above, this template library was composed of visual images of various vehicles from different perspectives, and each picture in the template library could be replaced according to driving condition, thereby expanding the application range of the template matching algorithm and optimizing the matching accuracy.

- Classifier-based Methods

The classifier-based methods establish an image classifier to distinguish vehicle targets versus non-vehicle targets in the candidate area. A large number of labeled positive (vehicle) and negative (non-vehicle) samples are used to train a classifier to learn the characteristic of the vehicle. This approach consists of two steps: feature extraction and object classification.

**(a)**  **Feature Extraction** 

In general, feature extraction refers to the process of converting the training samples into a feature vector that satisfies the input of the classifier. In order to achieve better classification results, the design and selection of features are particularly important. A fine feature should include most of the appearance of the vehicle, and should be as simple as possible to improve training efficiency. Commonly used feature extraction methods include HOG feature, Gabor filter, PCA, Haar feature, SIFT and SURF feature. 

HOG refers to Histogram of Oriented Gradient, which was first proposed in [138] for pedestrian detection, and then gradually used in the related work of vehicle detection. Most of the current research are devoted to optimizing conventional HOG in order to improve its calculation efficiency and detection accuracy.

In [139], the vehicle feature was extracted based on traditional HOG methods for classification training. In [140], the performance of three feature extraction methods of CR-HOG, H-HOG and V-HOG in vehicle detection were compared, and it was found that V-HOG has the best overall effect, compared with the conventional HOG method, the calculation efficiency was improved with the reducing of accuracy. The results of this literature were further verified in [141]. In [142], the accuracy of the V-HOG was optimized by constantly adjusting its parameters from experimental results. In [143], the calculation efficiency was improved by reducing the dimensions of the extracted feature vector based on the traditional HOG method. Since the traditional HOG method can only calculate the gradient features in both horizontal and vertical directions, in [144], Compass-HOG was designed to expand the direction dimension of image gradient calculation to reduce information loss and improve accuracy. In [145], 2D-HOG is designed to deal with the problem of resolution change of input image, and the accuracy was also improved compared with HOG.

The principle of the Gabor filter is to perform Fourier transform in a specific time window of the image, which can better extract the straight line and edge features of different directions and scales in the image. This method is very similar to the response of human vision to external stimuli, and can effectively extract image frequency-domain information, however, it has a high computing cost.

In [146], a parameter optimization method based on a genetic algorithm was designed for the Gabor filter to extract vehicle features. In [147,148], the Log–Gabor filter was designed to compensate for its amplitude attenuation in the process of processing natural language images [149] to achieve better image frequency-domain information extraction characteristics in vehicle detection. In [150], vehicle features in the night environment were extracted by the Gabor filter, and the filter parameters were adjusted through experiments.

PCA refers to Principal Component Analysis, converting the relevant high-dimensional indicators into low-dimensional indicators to reduce the computing cost with as less as possible loss of the original data.

In [151], PCA was used to extract vehicle features and SVM was trained to classify the generated ROIs, which can identify vehicles in front view and rear view at the same time. Since the traditional PCA extracted one-dimensional feature vectors of the image, the vector dimension is large with high computing cost, in [152], the vehicle feature extraction was realized by 2D-PCA combined with a genetic algorithm, nevertheless, the image pixel of the dataset used by the author is relatively low, optimizing computation efficiency by reducing the pixel was not representative. Thus in [153], the pixel of datasets was improved, 2D-PCA was combined with a genetic algorithm, fuzzy adaptive theory and self-organizing mapping for vehicle identification. In [154], features were extracted by HOG and dimensionality was reduced by PCA to reduce the amount of computation.

The Haar feature is based on the integral map method to find the sum of all the pixels in the image, which was first applied to face recognition in [155], Haar features include edge features, straight-line features, center features and diagonal features. The Haar feature is suitable for extracting edge features and symmetry features of vehicles, with a high computational efficiency to better meet the real-time requirements of vehicle detection.

In [156], the Haar feature was combined with a 2D triangular filter to achieve feature extraction. In [157,158], the Haar feature was introduced into LBP to realize vehicle detection through statistical image texture features, In [70], detection of frontal vehicles was based on the Haar feature, and active learning was then carried out to realize the detection of occluded vehicles. In [159], the Haar feature was used in infrared images combined with the maximum entropy threshold segmentation method to achieve vehicle detection.

SIFT refers to Scale-Invariant Feature Transform. It was proposed in [160], generating features by extracting key points in the image and attaching detailed information.

In [161], feature points were extracted based on SIFT, and feature vectors near feature points were extracted using the implicit structural model (ISM) to train SVM to detect vehicles. Due to the slow computing speed of the traditional SIFT method, in [162] vehicle feature was extracted by the Dense-SIFT method, so as to realize the detection of remote moving vehicles and improve the computing efficiency. In [163], the color invariant “CI-SIFT” was designed to enable it to have good characteristics when detecting vehicles of different colors. The author first recognized the body color through HSV color space, and then extracted the features through CI-SIFT, finally, vehicle detection was realized based on the matching algorithm.

SURF feature is the optimization and acceleration of SIFT feature. In [164,165], the symmetric points of vehicles were extracted based on SURF features to realize vehicle detection. By combining Haar features and SURF features, the real-time performance of this algorithm was improved by combining good robustness of SURF features and fast calculation speed of Haar features in [166]. In order to further improve computational efficiency, SURF characteristics and the BOVW model were combined to realize the detection of front and side vehicles in [167].

The comparison of different feature extraction methods is shown in Table 5.

**(b)**  **Object Classification** 

The purpose of object classification step is to choose or design a classifier according to the extracted features. The most commonly used classifiers for hypothesis verification are SVM and AdaBoost, related works are listed in Table 6.

### 3.2. Deep-Learning Based Methods

The aforementioned two-stage method includes two steps: HG and HV to form two-stage detector. In general, deep-learning based methods refer to designing a single-stage detector which does not need to extract ROIs from the image through training a neural network, but directly considers all regions in the image as region of interest, the entire image is taken as input, and each region is judged to verify whether it contains vehicles to be detected. Compared with the two-stage method, this method omits ROIs extraction and achieves a much faster processing speed, which is suitable for scenes with high real-time requirements, however, the detection accuracy is relatively low and the robustness is poor. In addition, there are also two-stage detectors designed based on deep learning methods, such research will be discussed together in this section.

#### 3.2.1. Two-Stage Neural Network

Generally speaking, the two-stage neural network is composed of region proposal stage and region verification stage, where the region proposal stage aims to generate candidate regions, while the region verification stage is carried out to train a classifier based on features generated by the convolution process to determine whether there is a true vehicle in candidate regions. With the development of deep learning technology, it has been widely used in various fields for its highly nonlinear characteristic and good robustness. Classical and recent neural networks are summarized in Table 7 and Table 8.

#### 3.2.2. One-Stage Neural Network

Training a one-stage neural network to achieve vehicle detection have emerged in recent years, typical single-stage detectors include the YOLO series and SSD. YOLO was proposed by [180] and it was the first single-stage detector in the field of deep learning. The framework of YOLO was a deep convolutional neural network (DCNN) and full convolutional neural network (FCNN), where DCNN was used to extract image features and greatly reduce its resolution to improve computational efficiency, FCNN was adapted for classification. Although YOLO has fast detection speed, it sacrificed detection accuracy. Thus in [181], SSD was proposed to solve the limitations of YOLO, which increased the resolution of the input image before extracting image features, thereby improving the detection accuracy, and also allowing to detected objects with a different scale. Subsequent YOLO-based improved networks include YOLO9000 (YOLOv2) [182], YOLOv3 [183], and recently optimized detection efficiency of YOLOv4 [184] and YOLOv5 [185]. Typical one-stage networks are summarized in Table 9.

In addition to the typical networks mentioned above, there are also many scholars who have improved original networks and designed new networks on this basis. See Table 10 for related work.

## 4. Vehicle Detection: Lidar-Based Vehicle Methods

Although vision-based vehicle detection methods are popular among UGVs, the lack of depth information makes it difficult to obtain vehicle position and attitude information. Therefore, three-dimensional detection methods are significant to be designed to achieve better scene understanding and communication with other modules such as planning and decision making, furthermore, they are also important for vehicle-to-everything (V2X) in ITS application [192]. Lidar is a good choice to effectively make up for the shortcomings of vision methods in vehicle detection, related approaches can be divided into four categories: classical feature extraction methods and learning-based approaches including projection methods, voxel methods and point-nets methods. Characteristics of each method and related learn-based methods are summarized in Table 11 and Table 12.

### 4.1. Feature Extraction Methods

Classical feature extraction methods for Lidar mainly refer to extracting various types of features by processing point clouds, such as lines extracted by Hough Transform, planes fitted by RANSAC. In the field of vehicle detection, vehicle geometric feature and vehicle motion feature are usually extracted from point clouds to achieve vehicle detection.

#### 4.1.1. Vehicle Geometric Feature

Vehicles show various types of geometric features in point clouds, such as planar, shape, and profile. Therefore, vehicle detection can be realized by extracting geometric features in Lidar point clouds.

In [193], a 3D occupancy grid map was first constructed through octree, then a list of the grids whose states were inconsistent between the current and previous scan was maintained as potential areas of objects, finally, the shape ratio feature of potential areas was extracted to achieve vehicle detection. However, this extracted shape feature was not robust for occluded vehicles. In [194], a Bayesian approach for data reduction based on spatial filtering is proposed that enables detection of vehicles partly occluded by natural forest, the filtering approach was based on a combination of several geometric features including planar surface, local convex regions and rectangular shadows, finally features were combined into maximum likelihood classification scheme to achieve vehicle detection. In [195], profile features were first extracted under polar space as the input of the subsequent detection scheme, then an online unsupervised detection algorithm was designed based on Gaussian Mixture Model and Motion Compensation to achieve vehicle detection. In [196], vehicle shape features were extracted approximately through non-uniform rational B-splines (NURBS) surfaces to achieve vehicle detection and tracking. In [197], the shape features of vehicles were predefined by constructing a CAD point clouds model of vehicles, then point clouds registration was carried out to realize vehicle detection and tracking. Results showed very good performance in detecting and tracking single vehicles without occlusions.

#### 4.1.2. Vehicle Motion Feature

The movement of vehicles in the environment will cause inconsistencies in Lidar point clouds of different frames. Thus, positions that may contain moving vehicles can be generated by extracting motion features in point clouds.

In [198], vehicle motion features were extracted from the continuous motion displacement, and are represented by rectangular geometric information on the 2D grid map. The algorithm was implemented on “Junior” which won second place in the Urban Grand Challenge in 2007. In [199], motion features were extracted by estimating Lidar flow from two consecutive point clouds, then FCN was trained to generate 3D motion vectors of moving vehicles to achieve vehicle detection as well as motion estimation.

Considering that it is a great challenge to detect vehicles that are far from Lidar because of the sparse point clouds. In [200], a dynamic vehicle detection scheme based on a likelihood-field-based model with coherent point drift (CPD) is proposed to achieve vehicle detection. Firstly, dynamic objects were detected through an adaptive threshold based on distance and grid angular resolution, then vehicle pose was estimated through CPD, finally, vehicle states were updated by Bayesian filter. Results showed that the proposed algorithm especially increased the accuracy in the distance of 40~80 m.

### 4.2. Projection Methods

Since vehicle detection in 2D vision images is a hot topic due to the various kinds of methods as well as the high availability of datasets, projection methods are put forward to transform Lidar point clouds into 2D images with depth and attitude information that can be processed via 2D detection methods. Related approaches can be divided into three categories including spherical projection, front-view projection and bird-eye projection on the basis of the representation of Lidar point clouds data.

#### 4.2.1. Spherical Projection

Spherical projection refers to projecting point clouds to a spherical coordinate system. The information contained in each point includes azimuth, elevation and distance from the Lidar scanning center.

Related work mainly focused on deep learning methods after projecting point clouds to the spherical image. In [201], “SqueezeSeg” was trained based on CNN to achieve detection after completing the point cloud projection. In [202], “PointSeg” was trained also based on CNN.

#### 4.2.2. Front-View Projection

Front-view projection refers to projecting the point clouds into the camera plane (similar to the depth image generated by a stereo camera). However, this kind of approach would generate numerous empty pixels at long-distance from Lidar due to the sparse distribution of point clouds. Thus in [203], a high-resolution image was constructed through a bilateral filter, results showed that the point cloud density of vehicles, pedestrians, etc., in the image had increased to a certain extent to optimize the overall resolution.

In [204], after completing the front-view projection, FCNN was performed for vehicle detection. In [205], ROIs were generated based on the DBSCAN algorithm, and ConvNet was trained for verification. Since the characteristics of the point cloud information for variant types of objects are different due to the measurement distance, angle and material of the object, in [206], Lidar echo intensity information was fused on the basis of [205] to firstly generate “sparse reflection map” (SRM), and points were connected into non-coincident triangles to establish “dense reflection map” (DRM), finally “ConvNet” was trained for faster vehicle detection compared with [205].

Some scholars also perform detection by fusing camera data. In [207], a non-gradient optimizer was carried out to fuse camera Lidar data, project Lidar point cloud data into a depth map, and establish Faster R-CNN for target detection, in [208], point clouds were projected into plane images and echo Intensity map, ROIs were generated from the camera image, and then the active learning network is trained for verification.

#### 4.2.3. Bird-Eye Projection

Bird-eye projection refers to projecting the point clouds into the top-view plane that is able to directly provide size and position information of objects to be detected. The bird-eye view can be further divided into three types [211]: height map generated by computing the maximum height of the points in each cell, intensity map generated according to the reflectance value of the point which has the maximum height in each cell, density map generated based on the number of points in each cell.

Deep learning methods are still popular in current research. In [209], multiple height maps, intensity maps and density maps were established, vehicle detection was then implemented based on CNN. Similar approaches included “Birdnet” in [210], “Complex-YOLO” in [211,212].

In [213] a three-channel bird’s eye view was established based on the maximum, median, and minimum height values of all points in the grid, which enable the network used for RGB image detection to be transferred for Lidar detection, and then RPN is used to realize vehicle detection with posture information. In [214], a heightmap and an intensity map were generated only considering the point clouds with maximum height in each grid, and “YOLO-3D” was proposed for vehicle detection. In [215], Lidar bird’s-eye view was fused with camera image, and then CNN with “coefficient non-uniform pooling layer” was put forward for vehicle detection. In [216], a density map was first generated to predefine a calculation area, a “PIXOR” network was then designed based on CNN for vehicle detection. In [217], a series of height maps through slices was generated, and features were extracted through RPN with classifies based on FCNN.

In addition to deep learning methods, in [218] stereo vision and 2D Lidar were integrated for vehicle detection. ROIs were generated based on bird’s-eye view established from 2D Lidar with depth information, then the similarity measurement with loss function evaluation of vehicle template is established for vehicle detection. In [219], point clouds were directly projected to the bird’s-eye view, vehicles were detected based on the edge and contour features, and the detection areas containing vehicles were trimmed according to vehicle size information to achieve optimization.

### 4.3. Voxel Methods

The voxel method decomposed the environmental space into numerous voxels, and points are allocated to the voxel grid at the corresponding position. In this way, objects to be detected can be represented as 3D voxel grid with their shape and size information.

In [220], point cloud was voxelized and vehicles were detected based on CNN. In [221], a “3D-FCN” was established for vehicle detection. The main idea was to take down sampling of voxel characteristics at 1/8 step length, and then deconvolution with phase synchronization length. In [222], monocular camera and Lidar was combined for vehicle detection. Firstly, the candidate regions were extracted from camera images based on “2D-CNN”, then the voxels in candidate area were matched and scored with the established three vehicle point cloud models (SUV, car and van), which were finally verified by CNN.

A more typical voxel method via neural network was “Voxelnet” designed in [223]. The main idea of this method was the designed “VFE layer” to characterize each Voxel, then objects could be detected through RPN. In [224], the “Second” network was designed based on “Voxelnet” to improve the processing capacity for sparse voxel grid, and a “angle loss regression equation” was designed to improve the detection performance of attitude angle. Subsequent improvements based on “Voxelnet” include “Part-A2” in [225] and “MVX-NET” in [226].

In addition to the deep learning method adopted by most researchers, in [227], a 3D occupancy grid map was generated after voxelization, and vehicles were detected by particle filter algorithm.

### 4.4. Point-Nets Methods

Compared with the projection method and the voxel method, point–nets method does not need to preprocess the point clouds. It directly regards the raw point clouds data as input to vehicle detection system with fewer points information loss, such approaches usually depend on an end-to-end deep-learning framework to process point clouds data.

The point-nets method was first proposed in [228]. The author designed a “PointNet” neural network to directly detect targets with the original point cloud of Lidar as input. The author then designed “PointNet++” [229] on the basis of “PointNet” to improve its ability of fine-grained identification (object subclass identification) to make it better applied in complex scenarios. It was pointed out in [230] that point clouds from Lidar were irregular and disordered, therefore applying direct convolution processing would cause shape information loss. Thus, an “X transformation” was first conducted for the processing of point clouds, and then “PointCNN” was established for vehicle detection. Other subsequent point-nets methods included “IPOD” in [231], “PointPillars” in [232] and “PointRCNN” in [233].

A fusion method of monocular camera and Lidar with limitation point cloud processing area was proposed in [179]. ROIs were first generated through the “RoarNet-2D” network. Then, the “RoarNet-3D” network was designed to detect vehicles from candidate areas and obtain the final attitude information of vehicles in order to lower the computing cost of point cloud processing.

## 5. Vehicle Detection: Radar-Based Methods

Radar has a wide range of applications in vehicle detection with higher cost performance, with the development of communication technology, automotive radar applications have played an increasingly critical role in intelligent transport system since it can obtain numerous types of information of the object (e.g., distance, relative speed, phase information), and is not affected by weather conditions. Therefore, co-existence between radars and UGVs has become more and more important. The commonly used radars for UGVs are millimeter-wave radar and ultrasonic radar (sonar). Both have similar working principles, therefore, this article only reviews the vehicle detection methods using millimeter-wave radar (MMW). The radar-based vehicle detection methods mainly include registration methods, learning-based methods, end-to-end methods and advanced radar-based imaging methods. Related works are summarized in Table 13.

### 5.1. Registration Methods

The essence of the registration method is the sensor fusion vehicle detection framework of MMW and vision sensors to achieve a better balance between detection accuracy and real-time performance. The vehicle position and speed information are firstly derived from MMW to initially generate ROIs, which are then registered by coordinate transformation with images to achieve joint vehicle detection.

In [234], MMW and monocular camera were fused for vehicle detection, MMW data were first transformed to the camera plane to jointly generate ROIs, and then verified based on DPM. In [235], vehicle contour features were used to verify ROIs after registration. In [189], MMW was used to extract feature points of vehicles, they were then transformed to the camera plane to jointly generate ROIs, and finally verified based on YOLOv2. In [236], the algorithm framework was similar to that of [189], however, ROIs were verified by HOG-SVM.

In [237], a stereo camera was equipped to detect side and nearby vehicles, while MMW was used to detect distant and longitudinal vehicles. The vehicle’s attitude and relative speed were estimated by MMW, and feature points were projected to the camera plane to realize jointly multi-directional vehicle detection.

### 5.2. Learning-Based Methods

Learning-based methods utilized for radar mainly include LSTM and Random Forest, this approach requires the establishment of a training set. Usually, the training data are clustered and calibrated first, then features are extracted and converted into feature vectors to input into the classifier.

In addition to vehicles, this method can also detect other road users such as pedestrians and bicycles. In [238,239], radar data were clustered based on the DBSCAN method, and results of random forest and LSTM in vehicle detection were compared. In [240], ROIs were extracted based on radar echo intensity, and LSTM was used to classify and track targets.

The above works all cluster radar data and convert it into feature vectors, then determines which type of target it belongs to (vehicles, pedestrians, bicycles, etc.), however, a different approach was carried out in [241]. After clustering the data, it directly judged the category based on the characteristics of the clustering points, and then LSTM was utilized to determine the correctness of classification (two-category classification); compared with traditional classification methods, the accuracy of this approach is improved by about 2%.

### 5.3. End-to-End Methods

The end-to-end methods directly use radar data as input to train a neural network for vehicle detection, whose principle is similar to that of “point-net methods” introduced in the above section. Due to the similarities between radar data and Lidar data, the design of the network often relies on the Lidar end-to-end framework.

In [242], radar data were directly used as input to PointNet++ for vehicle detection, while “PointNets” was used in [243]. In [244], “RTCnet” was established based on CNN with the input of the vehicle distance, azimuth and speed information collected by radar for vehicle detection.

### 5.4. Advanced Radar-Based Imaging Methods

The aforementioned vehicle detection methods are all based on the principle of radar echo to obtain distance, velocity and other types of information to achieve vehicle detection, however, the detected vehicles cannot be embodied or visualized. If the scene within UGVs’ detection range can be imaged by radar, the accuracy and scalability of the detection algorithm can be improved, and more complete environmental information can be obtained under different weather conditions. Advanced radar-based imaging methods has become a rapidly emerging technique, and it has great potential for improving the stability of UGVs.

In general, advanced radar-based imaging technology is usually applied in the field of aerospace. However, some related research is still carried out among vehicle detection in UGVs, and research about vehicle detection through radar-based imaging technology is summarized below.

High-resolution radar imaging can be achieved through SAR imaging technology. Using a suitable algorithm to generate images from radar data is the basis of applying advanced radar imaging technology to UGVs, algorithms about SAR imaging were reviewed in [245]. However, SAR data are inherently affected by speckle noise, methods related to reducing speckle noise for full polarimetric SAR image were briefly reviewed in [246]. In addition, real-time performance of SAR imaging is also crucial for the efficient operation of UGVs, SAR sparse imaging technologies that will help improve real-time performance were reviewed in [247].

In [248], a squint SAR imaging model was proposed via the backward projection imaging algorithm to perform high-resolution imaging for vehicle detection. In [249], Maximally Stable Extremal Region (MSER) methods were carried out to generate ROIs of vehicles, and a morphological filter was utilized to redefine ROIs; finally, the width-to-height ratio was used for verification to achieve vehicle detection in a parking lot. The same work was also carried out in [250], where the spectral residual was utilized to judge the postures of the vehicles, and vehicle detection was realized by PCA with SVM.

To make a better balance between image resolution and real-time performance, in [251], a hierarchical high-resolution imaging algorithm for FMCW automotive radar via MIMO-SAR imaging technique was designed for improving real-time performance while process imaging resolution, the algorithm was implemented in a UGV for roadside vehicle detection with a run time of 1.17 s/fps.

## 6. Vehicle Detection: Infrared-Based Methods

Commonly used vision-based vehicle detection algorithms are extremely susceptible to illumination conditions, the efficiency of the algorithm will greatly reduce especially at nighttime or under bad weather conditions. Therefore, the implementation of an infrared camera is crucial to compensate for vehicle detection under poor illumination conditions.

Some researchers used vision-based methods for vehicle detection in infrared images. In [252], edge features of vehicles in infrared images were extracted for vehicle detection. In [253], edge features were also extracted to generate ROIs, then, a vehicle edge template was established to achieve verification, the algorithm was embedded into FPGA and the running time reached 40 ms/fps. In [254], a polar coordinate histogram was established to extract vehicle features based on the polarization characteristics of vehicles in infrared images, with SVM implemented to classification. In [255], HOG was carried out to extract vehicle features, with supervised locality-preserving projections (SLPP) method to reduce dimensionality, and finally, an extreme learning machine was trained for classification.

It should be noted that the resolution of the infrared image is relatively low, consequently, accuracy feature extraction is difficult, some researchers first enhanced the contrast of infrared images in order to achieve better results. In [256], the contrast of infrared images was first enhanced, then ROIs were generated based on image saliency and average gradient method, finally, the confidence level was assessed to verify the ROIs. In [159], the contrast between the vehicle and the background was enhanced through top-hat transformation and bottom hat transformation, then vehicle features are extracted through the Haar method, and ROIs were generated through the improved maximum entropy segmentation algorithm which was finally verified by vehicle prior knowledge (vehicle size and driving position).

Two or more infrared cameras can also be equipped to form an “infrared stereo rig” to obtain depth information. In [51], two infrared cameras were used to form a stereo infrared vision, and a disparity map was generated for vehicle detection under different weather conditions.

With the development of artificial intelligence technology, some researchers have applied the deep learning framework for vehicle detection in infrared images. In [257], improved YOLOv3 was put forward for vehicle detection in infrared images. In [258], SSD was used with the adding of the “incomplete window” module to optimize the structure of datasets to solve the problem of vehicle missed detection.

## 7. Vehicle Detection: Event-Based Methods

Compared with traditional cameras, millisecond-level time resolution for event cameras makes it have powerful potential for detecting dynamic objects. Due to the low resolution of sensors, event cameras are currently used to detect small-sized targets. For example, in [259], a robot was used as a platform to detect small balls based on clustering and polar coordinate HOG transformation, in [260], a hierarchical model “HOTS” was established to recognize dynamic cards and faces. The application of event cameras for UGVs mainly focuses on SLAM and 3D reconstruction, and the generated 3D maps and models can intuitively represent the vehicles to be detected in the environment, but it is difficult to extract information from the maps or reconstructed models for subsequent planning and decision-making.

There has been little related research carried out with event cameras for vehicle detection. In [261], a time average histogram “HATS” was presented to extract vehicle features, with machine learning methods to achieve detection and classification. In [262], visual camera and event camera were fused, and an “SNN” was built based on CNN for vehicle detection under different illumination conditions. Although there are few relevant studies, it should be noted that event cameras have great potential for development in detecting moving vehicles.

## 8. Vehicle Detection: Sensor-Fusion Methods

Compared with separate sensors, vehicle detection with multi-sensor fusion can fuse the characteristics of different sensors to achieve higher accuracy, wider application range and stronger robustness, nevertheless, it will increase the complexity of models and algorithms, computing time, and the cost of sensors implemented on UGVs.

Sensor fusion needs to solve two main problems: Which sensors are needed to be fused? How does one distribute the work of different sensors? There are no conventional answers to these questions. Generally speaking, sensor fusion also needs to consider numerous details such as sensors calibration, sensors fusion level and fusion network. Here, we only summarize recent sensor-fusion-based methods for vehicle detection including Radar-Vision fusion and Lidar-Vision fusion methods, for detailed architecture and methods about sensor fusion can refer to [263]. Related works are summarized in Table 14.

## 9. Simulation Platform for Vehicle Detection

When a new algorithm is developed, it is usually difficult to directly verify its performance in UGVs for real scenarios. Since the instability and uncertainty of newly develop algorithm must be considered, which may cause danger and accident. Therefore, before testing the algorithm in a real scenario, preliminary tests in a simulation platform should be conducted to quickly find out the problems as well as improve the algorithm. The constructed simulated environment can help to shorten the development cycle, reducing development cost, increasing the safety of the test and constructing a variety of scenarios even under extreme conditions for testing in a simulated driving environment.

Testing in the simulation platform mainly includes three procedures. Firstly, a simulation scene should be set up, in which vehicles, pedestrians, buildings and roads in the real world are modeled on the platform including their appearance and dynamics model. Secondly, a sensor model should be established to convert the scene constructed into the data type received by the sensor. Finally, the algorithm is carried out for simulated tests. Here we introduce the commonly used simulation platforms in detail, for information about more comprehensive simulation platform can refer to Table 15.

### 9.1. Gazebo

Gazebo [271] is an open-source simulation platform mainly for robots based on the Robot Operating System (ROS). There are numerous interfaces between Gazebo and 3D modeling software, such as Solidworks and ProE, which facilitates the import of 3D models for UGVs. In the aspect of scene construction, a simulation environment can be built by placing geometry, but it is not suitable for building a complex driving environment. Sensors including Lidar, camera, GPS and IMU can be realized in Gazebo. In addition, the dynamic model of two to six-wheeled platform can be realized through a “differential drive plug-in”. The gazebo is usually used with Rviz to build a joint simulation environment, and then users can visualize the sensor detection results and platform movement process in Rviz. It should be noted Gazebo is mainly used for robotics and small UGVs, thus it is not suitable for simulation and verification of large outdoor UGVs.

### 9.2. Autoware

Autoware [272] is a simulation platform developed by a team from Nagoya University for autonomous driving basing on ROS. Works including SLAM, localization, object detection (vehicle, pedestrian, traffic light, etc.), path planning decision and motion control simulation can be realized in Autoware. This simulation platform integrates many mainstream algorithms such as YOLO, SSD and Euclidean clustering, which can be directly used, in addition, algorithms designed by users can also be verified based on this simulation platform. Last but not least, data collected from the real environment by the sensor can also be processed, and carry out the testing of various algorithms.

### 9.3. Udacity

Udacity [273] is developed based on the Unity3D engine, which mainly conducts simulation test for the deep learning algorithm for UGVs. This simulation platform is similar to a racing game, including training mode and automatic mode. In the training mode, users can manually control the vehicle to record data and train the designed deep learning model (the model can be built through C++ and python). Then, the trained model can be used to control the vehicle running in the automatic mode to evaluate the model.

### 9.4. Carla

Carla [274] is a simulation platform developed by Intel LABS and Toyota Research Institute for UGVs simulation in urban environments based on Unreal Engine 4. This platform is built with a number of urban scenes, including the numerous types of road, pedestrian and vehicle models, different weather conditions can also be configured, such as rain, snow, fog, noon and sunset, to test the effect of the algorithm under different conditions. Carla has powerful functions and good rendering effects, but it needs large running memory and high requirements for computer configuration.

### 9.5. AirSim

AirSim [275] is developed by Microsoft based on Unreal Engine 4 for UGVs and UAVs. Simulation scenes including city, countryside with better rendering effect is constructed in this platform. Moreover, AirSim has many types of interfaces that can be combined with C++, Python, Java and other programming languages. In particular, it performs well performance in verifying artificial intelligence methods such as machine learning and deep learning algorithm.

### 9.6. Apollo

Apollo [276] is developed by Baidu Company. It is equipped with algorithms in aspects of perception, planning, decision-making, etc., the data collected by users from sensors can also be tested through algorithms embed in Apollo. This platform is supported by cloud technology, thus high computing efficiency can be achieved by virtue of the powerful cloud computing capability.

### 9.7. Deepdrive

Deepdrive [277] is a simulation platform developed by The University of Berkeley, based on Unreal Engine. It also includes a variety of built driving scenarios, mainly for verifying related algorithms of deep learning.

## 10. Datasets for Vehicle Detection

Datasets are of great significance for the validation of designed algorithms. Datasets mainly include environmental information including vehicles, pedestrians, buildings, and more, collected by different sensors equipped on the platform under various driving conditions. With the development of sensors and computer technology, more and more research institutions share their collected and calibrated datasets to the network to achieve open source. Datasets related to vehicle detection technology are summarized in Table 16 below.

## 11. Summary and Prospect

UGVs have profound application prospects in both civil and military fields, thus have gradually become the focus of research in various countries. With the progress of the social economy, science and technology, UGV technology has made rapid progress. This paper first introduces the sensors commonly used in UGVs. Moreover, research of vehicle detection in the field of environmental perception is summarized for different sensors. Then, simulation platforms that can be applied to UGVs are described to facilitate the simulation test of the algorithm. Finally, datasets of UGVs are listed to verify the actual effect of the algorithm. The future research emphasis of vehicle detection technology for UGVs are forecasted on the following aspects.

### 11.1. Sensor-Based Anomaly Detection

The performance of sensors plays an important role in the efficiency and safe operation of UGVs. High accuracy and reliability sensors are very important for the construction of an environmental perception algorithm. If sensors are damaged during the runtime of UGVs no matter due to internal factors or external attacks, it will have an extremely adverse impact, even cause accidents, resulting in harming the economy and endangering lives.

Not only in the field of vehicle detection, as long as UGVs are in running status, but it is also necessary to carry out real-time monitor of sensor anomalies. Abnormal detection can be realized by monitoring the change range of sensor data or training the neural network [263], in addition, decision-making scheme should be further optimized to ensure that when any important sensor fails, UGVs can dock in the nearby safe area as soon as possible without affecting the operation of other road users. Therefore, how to efficiently design the sensor anomaly detection block and relevant decision-making scheme will be a focus of future research.

### 11.2. Multi-Mode Sensor Fusion for Vehicle Detection

In general, the application scenarios of vehicle detection based on a single sensor are limited, in order to make the algorithm applicable to more driving scenes, it is necessary to fuse detection methods of different sensors to realize the complementary advantages among each sensor. Sensor fusion will increase the complexity of the algorithm, and for simple driving scenes, high-precision detection can often be achieved with only a single sensor, in this case, sensor fusion methods will waste resources and affect the computing efficiency. Therefore, for different scenarios, different sensor detection schemes should be adopted, and sensor modes should be switched based on decision trees or other methods according to different working conditions so as to maximize resource utilization. Therefore, multi-mode sensor fusion for vehicle detection will be the focus of future research.

### 11.3. Special Vehicle Inspection

At present, most of the research works on vehicle detection focuses on the detection and identification of traditional vehicles, while there is little research devoted to special vehicle detection. In the civil field, the proper detection of ambulances, fire engines, police vehicles and other special vehicles is crucial to the rational decision for UGVs. In the field of military, there are various types and styles of ground vehicles, and the correct detection of different types of vehicles can promote the information acquisition of the battlefield and the correct issuance of operational instructions for UGVs. Therefore, how to construct the feature database and datasets to realize the efficient detection of special vehicles will be the focus of future research.

### 11.4. Vehicle Detection under High Speed

The high-speed running of UGVs is an important guarantee for its high operating efficiency. If the driving speed of the vehicle is increased, it is highly possible that the algorithm will not be able to process the environmental information in a good real-time performance, which will lead to wrong environmental perception and affect the security of UGVs. If the speed of the vehicle is slow, the efficiency of reaching the target position and achieving the expected goal will be reduced. Therefore, how to improve the real-time performance of the algorithm and ensure that UGVs can realize vehicle detection in the process of the high-speed driving condition is significant, which will become the research focus in the future.

## Figures and Tables

**Table 1:** Information for Different Exteroceptive Sensors.

Sensors	Affecting Factor		Color Texture	Depth	Disguised	Range	Accuracy (Resolution)	Size	Cost
	Illumination	Weather							
Lidar	-	√	-	√	Active	200 m	Distance accuracy: 0.03 m Angular resolution: 1.5°	Large	High
Radar (Long Range)	-	-	-	√	Active	250 m	Distance accuracy: 0.1 m~0.3 m Angular resolution: 2°~5°	Small	Medium
Radar (FMCW 77 GHz)	-	-	-	√	Active	200 m	Distance accuracy: 0.05 m~0.15 m Angular resolution: about 1°	Small	Very Low
Ultrasonic	-	-	-	√	Active	5 m	Distance accuracy: 0.2 m~1.0 m	Small	Low
Monocular Camera	√	√	√	-	Passive	-	0.3 mm~3 mm (Different fields of view and resolution have different accuracy)	Small	Low
Stereo Camera	√	√	√	√	Passive	100 m	Depth accuracy: 0.05 m~0.1 m Attitude resolution: 0.2°	Medium	Low
Omni-direction Camera	√	√	√	-	Passive	-	Resolution (Pixels): can reach 6000 × 3000	Small	Low
Infrared Camera	-	√	-	-	Passive	-	Resolution (Pixels): 320 × 256~1280 × 1024	Small	Low
Event Camera	√	√	-	-	Passive	-	Resolution (Pixels): 128 × 128~768 × 640	Small	Low

Note: The range of cameras except for depth range of stereo camera is related to operation environmental thus there is no fixed detection distance.

**Table 2:** Summary of hypothetical generation (HG) methods for monocular vision.

Methods		Literature	Pros	Cons
Appearance-Based Methods	Color	[53,54,55,56,57,58]	Low computing cost; Easy to implement; Color characteristics are generally obvious	Easily affected by illumination condition and shadow
	Edge	[59,60,61,62,63,64]	Low computing cost; Easy extraction of vehicle edge features.	Easily affected by other objects with obvious edge feature; Difficult to choose a suitable threshold
	Corner	[65,66]	High detection accuracy; Easily locate vehicle to be detected in the image.	Hard to apply in complex environments
	Symmetry	[67,68,69,70]	High detection accuracy; Highly symmetrical for common vehicles	High computing cost; Suitable for ahead and behind vehicle detection, bad performance for others viewing angle
	Texture	[71,72,73]	High detection accuracy	Hard to apply in complex environments; Easily affected by shadow
	Shadow	[74,75,76,77]	Low computing cost	Easily affected by illumination condition and shape of shadow; Difficult to choose and optimize threshold
	Lights	[78,79,80,81,82,83,84]	Better performance in the night environment	Easily affected by street lights and other non-vehicle lights
	Features Fusion	[62,69,85,86,87]	High detection accuracy; High robust and reliable	High computing cost; Complex algorithm structure;
Motion-Based Methods	Frame Difference	[88,89,90,91,92]	Fast detection speed and good real-time performance; Not easily affected by illumination condition	Impossible to detect stationary vehicles; Difficult to detect low-speed vehicles
	Background Modeling	[93,94,95,96,97,98,99]	Easy to implement; good real-time performance	Difficult to build background models for complex scenes; Background update is challenging
	Optical	[100,101,102,103,104]	Robust; Suitable for real-time monitoring of long video streams	Easily affected by illumination condition; Difficult to detect High-speed vehicles

**Table 3:** Summary of HG methods for stereo vision.

**Methods**	**Literature**	**Pros**	**Cons**
IPM	[105,106,107,108,109,110,111]	Low computing cost; Simple and mature algorithm	Vulnerable to road conditions including off-road and uneven road
Disparity Map	[112,113,114,115,116,117,118,119,120,121,122,123,124]	High detection accuracy; Easy to obtain depth information	High computing cost; Low resolution for planes with similar shapes
Optical Flow	[125,126,127]	Same with monocular camera	Same with monocular camera

**Table 4:** Summary of Fusion methods for HG.

Year	Literature	Features for Fusion	Datasets	Accuracy	Time (ms/fps)	Hardware	Adaption
2013	[62]	edge; shadow	100 images consist of downtown and highway	70%	-	Intel core i5; 2 GB RAM	Only good weather condition
2015	[85]	edge; texture	PASCAL VOC	About 80%	-	-	Better performance in detecting occluded vehicles
2015	[86]	edge; corner; lights; symmetry	iROADS	95.1%	40 ms	Core i5 2.7 GHz; 8 GB RAM	Daytime; nighttime; rainy; snowy
2018	[87]	color; texture	KITTI	89.91%	170 ms	Nvidia Titan X	Occluded vehicles in complex traffic environment
2019	[69]	edge; symmetry	Own dataset	94%	10 ms	Intel core i5	Vehicle without occlusion in simple traffic environment

**Table 5:** Summary of feature extraction methods for hypothetical verification (HV) stage.

Methods	Literature	Pros	Cons
HOG	[139,140,141,142,143,144,145]	Good optical and geometric invariance; High feature extraction accuracy	High computing cost
Gabor	[146,147,148,150]	Similar to the response of human vision to external stimuli; Effectively extract image frequency domain information	High computing cost
PCA	[151,152,153,154]	Feature vector dimension can be effectively reduced to improve calculation efficiency	How to effectively avoid information loss when reducing feature vector dimension remains a challenge
Haar	[156,157,158,159]	Various forms of extractable features; Low computing cost	How to select relevant feature templates to extract features for different scenarios remains a challenge
SIFT	[161,162,163]	Good scale invariance; Good local stability and scalability	High computing cost
SURF	[164,165,166,167]	Optimized computational efficiency compared to SIFT features	Search accuracy is reduced compared to SIFT feature

**Table 6:** Summary of related works for classifiers of HV stage.

Year	Literature	Feature	Classifier	Datasets	Accuracy	Time (ms/fps)	Hardware	Adaption
2009	[151]	PCA	SVM	PASCAL VOC; INRIA	94.93%	—	—	Vehicles without occlusion in simple traffic environment
2013	[139]	HOG	SVM	Own dataset	96.87%	40 ms	Core i5 2.67 GHz	Vehicles under various illumination condition
2017	[141]	HOG	SVM	GTI	98.61%	50 ms	—	Vehicles without occlusion in simple traffic environment
2018	[150]	Gabor	SVM	Own dataset	92.87%	—	—	Daytime; nighttime
2018	[158]	Haar	Adaboost	GTI	90.10%	—	Core i5 1.80 GHz; 4 GB RAM	Vehicles without occlusion in highway
2019	[145]	HOG +PCA	SVM	UIUC	99.28%	61 ms	CPU 2.9 GHz; 8 G RAM	Vehicles with multi-view

**Table 7:** Summary and characteristic of classical two-stage neural network.

Year	NN	Literature	Region Generation	Classifier	Comment
2012	CNN	[168]	Sliding Windows	SVM	Images were divided into different ROIs, and then classified after feature extracting through convolution.
2014	RCNN	[169]	Selective Search	SVM	Similar with CNN except that region generation is achieved through selective search.
2014	SPPNet	[170]	Selective Search	SVM	Convolution process directly on the original image and then extract ROIs through Selective Search.
2015	Fast-RCNN	[171]	Selective Search	SoftMax	ROI polling is different from SPPNet.
2017	Faster-RCNN	[172]	RPN [173]	SoftMax	Region generation is achieved through RPN.

**Table 8:** Summary of related works for recent two-stage neural network for vehicle detection.

Year	Literature	Network	Dataset	Accuracy	Time (ms/fps)	Hardware	Adaption
2018	[87]	FMLA-CNN	KITTI	88.83%	170 ms	NVIDIA TitanX	Vehicles with occlusion under various illumination condition
2018	[174]	Mobile-Net	Own dataset	91.59%	66 ms	NVIDIA TX2	Vehicles detection for 360° FOV in nighttime
2018	[175]	extraCK	KITTI	82.46%	30 ms	Inter Core i7 2.70 GHz	Vehicles with occlusion in simple traffic environment
2018	[176]	MFR-CNN	KITTI; PASCAL VOC	84.30%	105 ms	NVIDIA TitanX	Vehicles with multi-view under various traffic environment
2019	[177]	SINet	KITTI	89.21%	110 ms	NVIDIA TitanX	Vehicles under sparse and crowded highway environment
2019	[178]	CNN-LSTM	UC Merced	96.10%	—	—	Vehicles detection as well as tail lights recognition
2019	[119]	Faster-RCKK	KITTI	91.20%	200 ms	Two Inter Xeon	Vehicles at long distance under poor illumination condition
2019	[179]	RoarNet	KITTI	84.25%	65 ms	NVIDIA TitanX	Vehicles under various traffic environment

**Table 9:** Summary and characteristic of classical one-stage neural network.

Year	NN	Literature	Comment
2016	YOLO	[180]	Images were directly divided into defined number of grids, and then bounding box and category are predicted through a neural network.
2016	SSD	[181]	Allow to detect different objects with different scales compared with YOLO.
2017	YOLOv2	[182]	Better prediction, faster, and enable more type of objects to be detected.
2018	YOLOv3	[183]	Detection speed and accuracy can be balanced by changing the size of the network structure; FPN was implemented to achieve multi-scale prediction.
2020	YOLOv4	[184]	Network can be better used in practice and easier to train.
2020	YOLOv5	[185]	Faster detection speed with lightweight network.

**Table 10:** Summary of related works for recent one-stage neural network for vehicle detection.

Year	Literature	Network	Dataset	Accuracy	Time (ms/fps)	Hardware	Adaption
2018	[186]	MB-Net	KITTI	80.01%	19 ms	NVIDIA TitanX	Vehicles with occlusion under various traffic environment
2019	[187]	EZ-Net	Own Dataset	75.60%	7.14 ms	NVIDIA TitanX	Vehicles detection with panoramic image at both daytime and nighttime
2019	[188]	BS3D	KITTI	84.80%	21.88 ms	NVIDIA TitanX	3D regression BB for vehicles under various traffic environment
2019	[189]	YOLO with MMW	PASCAL VOC	90.90%	66.67 ms	—	Vehicles under weather condition (sunny, foggy, cloudy)
2019	[190]	Dense-ACSSD	BSD 100 K	84.02%	28.57 ms	GTX 1080Ti	Vehicles under crowded environment at both daytime and nighttime
2020	[191]	MSI-OHEM	PASCAL VOC	85.35%	15.63 ms	NVIDIA DriverPX2	Both car and bus can be detected under simple and moderate traffic environment

**Table 11:** Research of vehicle detection with Lidar.

Methods		Literature	Pros	Cons
Feature Extraction Methods		[193,194,195,196,197,198,199,200]	Good interpretability; High real-time performance	Poor robustness to changing environments
Projection Methods	Spherical	[201,202]	Point cloud get denser after transformation	Difficult to achieve sensor fusion
	Plane	[203,204,205,206,207,208]	Convince for data fusion with camera images	Empty pixels may be produced at distant location due to sparse point cloud.
	Bird-Eye	[209,210,211,212,213,214,215,216,217,218,219]	Directly provide the location and size information of the object	Sparse point cloud at distant location may cause error detection
Voxel Methods		[220,221,222,223,224,225,226,227]	Original 3D data information can be retained	Empty voxel grids are generated due to sparse and uneven distribution of point cloud
Point-Nets Methods		[179,228,229,230,231,232,233]	Simple and fast; No hard demand for point cloud pre-processing	Usually a long network training period

**Table 12:** Summary of related works for learning-based vehicle detection using Lidar.

Methods	Network	Dataset	Accuracy	Time (ms/fps)	Hardware	Adaption
Spherical Projection Methods	SqueezeSge [201]	GTA-simulated	69.6%	8.7 ms	NVIDIA TitanX	Vehicles under simple traffic environment
	PointSeg [202]	KITTI	74.8%	11 ms	GTX 1080Ti	Vehicles with occlusion under simple traffic environment
Front-View Projection Methods	DepthCN [205]	KITTI	56%	230 ms	GTX 1080; 64 GB RAM; Hexa core 3.5 GHz	Vehicles with occlusion under complex traffic environment
	ConvNets [206]	KITTI	61.14%	49 ms	GTX 1080; 64 GB RAM; Hexa core 3.5 GHz	Vehicles with occlusion under complex traffic environment
	Faster-RCNN [207]	KITTI	87.9%	250 ms	GTX graphics card; Intel Xeon processor	Vehicles under moderate traffic environment
Bird-Eye Projection Methods	BirdNet [210]	KITTI	67.56%	110 ms	GTX graphics card	Vehicles under various traffic environment
	YOLO-3D [214]	KITTI	75.3%	25 ms	-	Vehicles at long distance under moderate traffic environment
	PIXOR [216]	ATG4D	73.3%	100 ms	-	Vehicles mainly in front view under moderate traffic environment
	FCNN [217]	KITTI	65.89%	72 ms	NVIDIA TitanX	Vehicles with multi-view at long distance under moderate traffic environment
Voxel Methods	Voxelnet [223]	KITTI	65.46%	33 ms	NVIDIA TitanX	Vehicles under various traffic environment
	Second [224]	KITTI	76.48%	50 ms	GTX 1080	Vehicles under various traffic environment
	Part-A2 [225]	KITTI	79.47%	-	NVIDIA Tesla V100	Vehicles under various traffic environment
	MVX-NET [226]	KITTI	72.7%	-	-	Vehicles at long distance under various traffic environment
Point-Nets Methods	IPOD [231]	KITTI	76.4%	-	-	Vehicles under various traffic environment
	PointPillars [232]	KITTI	74.99%	16 ms	GTX 1080Ti	Vehicles under various traffic environment
	PointRCNN [233]	KITTI	78.63%	-	-	Vehicles under moderate traffic environment
	RoarNet-3D [179]	KITTI	74.29%	20 ms	NVIDIA TitanX	Vehicles mainly at long distance under moderate traffic environment

**Table 13:** Research of vehicle detection with Radar.

Methods	Literature	Pros	Cons
Registration Methods	[189,234,235,236,237]	Strong robustness to weather and illumination condition	Low detection accuracy under complex environment; Additional preparations such as coordinate transformation need to be carried out
Learning-Based Methods	[238,239,240,241]	High detection accuracy	Long training period; Low real-time performance under a complex environment.
End-to-End Methods	[242,243,244]	High real-time performance; Simple algorithm framework	Long training period; Poor interpretability
Advanced radar-based Imaging Methods	[245,246,247,248,249,250,251]	Environmental information can be obtained intuitively under different illumination and weather condition	How to achieving better compatibility with UGVs still remains challenges; Resolution and real-time performance still need to be improved

**Table 14:** Summary of sensor-fusion based methods for vehicle detection.

Fused Sensors	Literature	Works for Sensors	Fusion Characteristic
Radar- Vision	[189,234,235,237,264]	ROIs are generated based on radar and camera respectively and then matched.	Various information of detected target can be obtained. Strong robustness to weather and illumination condition. Low hardware cost [265]. Widely used in vehicle detection.
	[150,266,267,268,118]	ROIs are generated by radar first and then verified by vision methods.	
Lidar- Vision	[207,218,269]	Lidar data are projected into a specific view, then fused with ROIs generated by vision methods.	Various information of detected target can be obtained. Strong robustness to illumination condition. High hardware cost and high computing cost.
	[270]	Lidar data are segmented with edge feature extracted from vision image to achieve fused detection.	
	[179]	Deep-Learning based NN are trained for both Lidar and vision.	

**Table 15:** Summary of simulation platform for UGVs.

Simulation Platform	Current Version	License	Operating System	Usage	Support Language
AirSim (Microsoft)	June. 2018 vl .2	Open source (MTI License)	Linux, Windows	Drone and car simulation 3D visual environment HIL controller support	C++, C#, python, and java
ASM Traffic (dSpace)	2017	Commercial	N/A	DJL traffic environment simulation for ADAS controllers	N/A
CARLA	Jul. 2018 v0.9.0	Open source (MTI License)	Linux (Ubuntu 16.04 or later)	3D urban environment Camera and sensor simulation	Python
CarMaker (IPG Automotive)	N/A	Commercial Free trial on demand	N/A	Virtual testing driving	N/A
DYNA4 (TESIS)	2017 V2.8	Commercial	Windows	Modular simulation SIL and SIL functional testing report and analysis generation	C/C++, Matlab/Simulink
Gazebo for ROS	Jan. 2018 v9.0.0	Open source (Apache 2.0)	Linux, Mac OS X, Windows	Robot dynamics simulation 3D visual sensor data generation	C++
(Simulator of) HankVirtual Env. Lab	N/A	Access on demand	N/A (Hardware platform)	Bicycling and pedestrian simulator	N/A
Legion for Aimsun	N/A	Commercial	(Aimsun Plug-in)	Integrated pedestrian and traffic simulation for traffic engineering and planning	N/A
OpenDaVINCI and OpenDLV	Sep. 2017 v4.16.0	Open source (GPLv2, LGPLv2)	POSIX-com patible OS, Windows	Environment visualization sensor model Autonomous driving	C++, Python
PELOPS (fka)	2011	Commercial	Linux	Traffic simulation combining sub-microscopic vehicle model and microscopic traffic model	N/A
PreScan (Tass)	2018 v8.5	Commercial Free trial on demand	Windows	Sensor simulation for ADAS HIL driving simulation	Matlab/Simulink
PTV Vissim	Vl0.0	Commercial Free trial available	Windows	Road junction geometry Public transport simulation Active traffic management	N/A
Racer	Aug. 2014 V0.9.0	Free for Non-commercial use	Linux, Mac OS X, Windows	3D car racing simulation High DOF car modeling	C++
SCANeR Studio (OKTAL)	Oct. 2017 vl.7	Commercial	Windows	Traffic scenario simulation Vehicle dynamics Autonomous driving	C++, Matlab/ Simulink
Sim IV(VTI)	N/A	Commercial	N/A (Hardware platform)	2-Axe driving simulator facility with 210° forward FOV	N/A
Speed Dreams	Dec. 2015 v2.2 Beta	Open source (GPL)	Linux, Mac OS X, Windows (32-bit)	3D car racing erg simulation (TORCS alternative) Simu V3 physics engine	C/C++
SUMO	Dec. 2017 v0.32.0	Open source (EPLv2)	Linux, Windows	Urban traffic flow simulation Vehicular communication	C++
TORCS	Mar. 2017 vl.3.7	Open source (GPLv2)	Linux, FreeBSD, Mac OS X, Open Solaris, Windows	3D car racing simulation Programmable AI for racing	C/C++
VDrift	Oct. 2014	Open source (GPLv2)	Linux, FreeBSD, Mac OS X, Windows	3D car racing simulation Driving physics	C++
V-Rep (Coppelia)	Feb.2018 v3.5.0	Commercial Free educational license possible	Linux, Mac OS X, Windows	Virtual robot simulator Robotic dynamics and kinematics Sensor simulation	C/C++, python, Matlab, Octave, Java and Lua
VTD (Vires)	May. 2018	Commercial	N/A	Driving simulation tool-chain Free data standards	N/A

**Table 16:** Summary of datasets for UGVs.

Dataset	Environment	Sensors	Format and Capacity	Content
Apollo	Expressway under various weather conditions	3 monocular color cameras Lidar with 32 layers; Velodyne-64 Lidar; real time kinematic GPS + IMU	ca 270 GB in total (172 GB available); jpg or png: image; txt: label; bin: Velodyne; HD F5: image; curvature	Raw data (training/validation/motation/test sets); annotations/label; benchmark; source code; demo video
BDDV	Various road/weather/ lighting conditions	Monocular color camera; sensors from a smart phone: GPS/IMU; gyroscope; magnetometer	+100;000 videos; 40 s each (+1.8 TB); mov: video jpg: image; Json: label; other formats to be found by checking the dataset	Raw data (training/ validation/test sets); annotations: 2D bounding box; lane marking; drivable area; pixel/instance-level segmentation
Ford	Downtown; loop closure; campus	Velodyne-64 Lidar; omnidirectional camera; 2 Riegl LMS-Q120 Lidars; Applanix +Trimble GPS; Xsens consumer IMU	ca 100 GB; mat: Velodyne scan; ppm: image; log: sensor data and timestamp; pcap: Velodyne stream; mat: calibration;	Raw data; Matlab and C code
JAAD	Mainly urban; a few rural roads; most daytime; occasional night; various weather conditions	Monocular color camera	347 videos; 5–15 s each; mp4; seq: video; vbb/tsv: textual annotation; xml: bounding box annotation	Videos; textual and bounding box annotations; bash script for splitting videos
Karlsruhe labeled objects	Urban; daylight	Monocular grayscale camera	631.2 MB (ca 1800 images with labels); png: image; mat: label	Images; object labels; object orientation
Karlsruhe stereo	Urban; rural; daylight	Stereo grayscale camera; GPS + IMU	20 sequences (0.2–1.4 GB each); png: image; txt: GPS + IMU data	Raw data; camera calibration
KITTI	Urban; rural; highway	2 monocular grayscale cameras; 2 monocular color cameras; Velodyne-64 Lidar; GPS + IMU	180 GB; png: image; txt: velodyne and GPS + IMU data; calibration; xml: bounding box label	Raw data; object annotation (3D bounding box); calibration; various benchmarks: Matlab and C++ code
Malaga	Urban; highway; loop closure	Stereo color camera; 3 Hokuyo UTM-30LX laser scanners; 2 Sick Lidars; GPS + IMU	15 sequences (+70 GB); txt: raw laser scan; GPS/IMU data; camera calibration; jpg: image; rawlog: own format binary; kml: google earth file to represent path	Raw data; C++ example code for parsing raw log files; demo videos; support for posting public messages by users
MVD	Various road/weather/light conditions	Cameras of different devices: mobile phones; tablets; action cameras; professional capturing rigs	25;000 images (25.6 GB); jpg; png: image	Raw data (training/validation/test sets);object annotations
Stanford	Urban; campus; intersections	Velodyne-64 Lidar; Applanix (GPS/IMU)	33 files (5.72 GB); tm: Velodyne and Applanix data (own format)	Raw data; background data without objects (training and testing sets); object labels; code in ROS package
Udacity	Sunny; overcast; daylight	Monocular color camera; Velodyne-32 Lidar; GPS + IMU	223 GB (10 h); png or jpg: image; log: GPS and vehicle motion; csv: label; ROSBAG	Videos; labels: vehicle; pedestrian; traffic lights; open source code; tools for ROSBAG files
CityScapes	Urban; daytime; good and medium weather condition; different seasons	Stereo color camera; GPS + IMU	16 cities (12.7 GB); png: image; txt: labels	Raw data; bounding box annotations of people; images augmented with fog and rain; 25,000 annotated images
H3d-HRI-US	Urban; various traffic condition	3 monocular color cameras; Velodyne-64 Lidar; GPS + IMU	csv: yaw; speed; GPS + IMU; txt: labels ply: point clouds	Raw data; bounding box label for only 3D detection and tracking
nuScens	Urban; various weather condition; various traffic condition	Lidar; 6 monocular color cameras; 5 radars; GPS + IMU	1000 scenes of driving; customized data format containing various sensor data	Raw data; detailed map information; 3D bounding boxes annotation for 23 classes

## References

[1] Bishop R. (2000). A survey of intelligent vehicle applications worldwide. Proceedings of the IEEE Intelligent Vehicles Symposium 2000 (Cat. No. 00TH8511).

[2] Li Z., Gong J., Lu C., Xi J. (2020). Importance Weighted Gaussian Process Regression for Transferable Driver Behaviour Learning in the Lane Change Scenario. IEEE Trans. Veh. Technol..

[3] Li Z., Wang B., Gong J., Gao T., Lu C., Wang G. (2018). Development and Evaluation of Two Learning-Based Personalized Driver Models for Pure Pursuit Path-Tracking Behaviors. Proceedings of the 2018 IEEE Intelligent Vehicles Symposium (IV).

[4] Li Z., Gong C., Lu C., Gong J., Lu J., Xu Y., Hu F. (2019). Transferable Driver Behavior Learning via Distribution Adaption in the Lane Change Scenario. Proceedings of the 2019 IEEE Intelligent Vehicles Symposium (IV).

[5] Lu C., Hu F., Cao D., Gong J., Xing Y., Li Z. (2020). Transfer Learning for Driver Model Adaptation in Lane-Changing Scenarios Using Manifold Alignment. IEEE Trans. Intell. Transp. Syst..

[6] Ma W., Qian S. (2021). High-Resolution Traffic Sensing with Probe Autonomous Vehicles: A Data-Driven Approach. Sensors.

[7] Chen Y., Zhang Y. (2014). An overview of research on military unmanned ground vehicles. Binggong Xuebao/Acta Armamentarii.

[8] Sivaraman S., Trivedi M.M. (2013). Looking at vehicles on the road: A survey of vision-based vehicle detection, tracking, and behavior analysis. IEEE Trans. Intell. Transp. Syst..

[9] Xia M.L., Tang T.B. (2015). Vehicle detection techniques for collision avoidance systems: A review. IEEE Trans. Intell. Transp. Syst..

[10] Li G.Z., Lu C., Gong J., Hu F. A Comparative Study on Transferable Driver Behavior Learning Methods in the Lane-Changing Scenario. Proceedings of the 2019 IEEE Intelligent Transportation Systems Conference (ITSC).

[11] Hu L.F., Cao D., Gong J., Xing Y., Li Z. (2019). Virtual-to-Real Knowledge Transfer for Driving Behavior Recognition: Framework and a Case Study. IEEE Trans. Veh. Technol..

[12] Li J., Zhan W., Hu Y., Tomizuka M. (2019). Generic tracking and probabilistic prediction framework and its application in autonomous driving. IEEE Trans. Intell. Transp. Syst..

[13] Li K.M., Zhang Q., Luo Y., Liang B.S., Yang X.Y. (2014). Review of ground vehicles recognition. Tien Tzu Hsueh Pao/Acta Electron. Sin..

[14] Li J., Yang F., Tomizuka M., Choi C. Evolvegraph: Multi-agent trajectory prediction with dynamic relational reasoning. Proceedings of the Neural Information Processing Systems (NeurIPS).

[15] Ciaparrone G., Sánchez F.L., Tabik S., Troiano L., Tagliaferri R., Herrera F. (2020). Deep learning in video multi-object tracking: A survey. Neurocomputing.

[16] Luo W., Xing J., Milan A., Zhang X., Liu W., Kim T.-K. (2020). Multiple object tracking: A literature review. Artif. Intell..

[17] Mozaffari S., Al-Jarrah O.Y., Dianati M., Jennings P., Mouzakitis A. (2020). Deep Learning-Based Vehicle Behavior Prediction for Autonomous Driving Applications: A Review. IEEE Trans. Intell. Transp. Syst..

[18] Rosique F., Lorente P.N., Fernandez C., Padilla A. (2019). A Systematic Review of Perception System and Simulators for Autonomous Vehicles Research. Sensors.

[19] Munir A.F., Rafique A., Ko Y., Sheri A.M., Jeon M. (2018). Object Modeling from 3D Point Cloud Data for Self-Driving Vehicles. Proceedings of the 2018 IEEE Intelligent Vehicles Symposium (IV).

[20] Javanmardi M., Gu Y., Kamijo S. Adaptive Resolution Refinement of NDT Map Based on Localization Error Modeled by Map Factors. Proceedings of the 2018 21st International Conference on Intelligent Transportation Systems (ITSC).

[21] Kraemer S., Bouzouraa M.E., Stiller C. Utilizing LiDAR Intensity in Object Tracking. Proceedings of the 2019 IEEE Intelligent Vehicles Symposium (IV).

[22] Chen T., Wang R., Dai B., Liu D., Song J. (2016). Likelihood-Field-Model-Based Dynamic Vehicle Detection and Tracking for Self-Driving. IEEE Trans. Intell. Transp. Syst..

[23] Patole S.M., Torlak M., Wang D., Ali M. (2017). Automotive radars: A review of signal processing techniques. IEEE Signal Process. Mag..

[24] Zhou S.Z., Zhao C., Zou W. A compressed sensing radar detection scheme for closing vehicle detection. Proceedings of the 2012 IEEE International Conference on Communications (ICC).

[25] Pech H., Nauth P.M., Michalik R. A new Approach for Pedestrian Detection in Vehicles by Ultrasonic Signal Analysis. Proceedings of the IEEE EUROCON 2019-18th International Conference on Smart Technologies.

[26] Wu T., Tsai P., Hu N., Chen J. Research and implementation of auto parking system based on ultrasonic sensors. Proceedings of the 2016 International Conference on Advanced Materials for Science and Engineering (ICAMSE).

[27] Krishnan P. (2018). Design of Collision Detection System for Smart Car Using Li-Fi and Ultrasonic Sensor. IEEE Trans. Veh. Technol..

[28] Mizumachi M., Kaminuma A., Ono N., Ando S. Robust Sensing of Approaching Vehicles Relying on Acoustic Cue. Proceedings of the 2014 International Symposium on Computer, Consumer and Control.

[29] Syed A., Morris B.T. SSeg-LSTM: Semantic Scene Segmentation for Trajectory Prediction. Proceedings of the 2019 IEEE Intelligent Vehicles Symposium (IV).

[30] Weber M., Fürst M., Zöllner J.M. Direct 3D Detection of Vehicles in Monocular Images with a CNN based 3D Decoder. Proceedings of the 2019 IEEE Intelligent Vehicles Symposium (IV).

[31] Dai X., Liu D., Yang L., Liu Y. Research on Headlight Technology of Night Vehicle Intelligent Detection Based on Hough Transform. Proceedings of the 2019 International Conference on Intelligent Transportation, Big Data & Smart City (ICITBS).

[32] Han B., Wang Y., Yang Z., Gao X. (2019). Small-Scale Pedestrian Detection Based on Deep Neural Network. IEEE Trans. Intell. Transp. Syst..

[33] Wang Q., Gao J., Yuan Y. (2018). Embedding Structured Contour and Location Prior in Siamesed Fully Convolutional Networks for Road Detection. IEEE Trans. Intell. Transp. Syst..

[34] Wang J., Zhou L. (2019). Traffic Light Recognition with High Dynamic Range Imaging and Deep Learning. IEEE Trans. Intell. Transp. Syst..

[35] Tian Y., Gelernter J., Wang X., Li J., Yu Y. (2019). Traffic Sign Detection Using a Multi-Scale Recurrent Attention Network. IEEE Trans. Intell. Transp. Syst..

[36] Li L., Liu Z., Özgïner Ü., Lian J., Zhou Y., Zhao Y. Dense 3D Semantic SLAM of traffic environment based on stereo vision. Proceedings of the 2018 IEEE Intelligent Vehicles Symposium (IV).

[37] Zhu K., Li J., Zhang H. Stereo vision based road scene segment and vehicle detection. Proceedings of the 2nd International Conference on Information Technology and Electronic Commerce.

[38] Yang W., Fang B., Tang Y.Y. (2018). Fast and Accurate Vanishing Point Detection and Its Application in Inverse Perspective Mapping of Structured Road. IEEE Trans. Syst. Mancybern. Syst..

[39] Doval G.N., Al-Kaff A., Beltrán J., Fernández F.G., López G.F. Traffic Sign Detection and 3D Localization via Deep Convolutional Neural Networks and Stereo Vision. Proceedings of the 2019 IEEE Intelligent Transportation Systems Conference (ITSC).

[40] Donguk S., Hansung P., Kanghyun J., Kangik E., Sungmin Y., Taeho K. Omnidirectional stereo vision based vehicle detection and distance measurement for driver assistance system. Proceedings of the IECON 2013—39th Annual Conference of the IEEE Industrial Electronics Society.

[41] Arnold E., Al-Jarrah O.Y., Dianati M., Fallah S., Oxtoby D., Mouzakitis A. (2019). A Survey on 3D Object Detection Methods for Autonomous Driving Applications. IEEE Trans. Intell. Transp. Syst..

[42] Wang S., Yue J., Dong Y., Shen R., Zhang X. Real-time Omnidirectional Visual SLAM with Semi-Dense Mapping. Proceedings of the 2018 IEEE Intelligent Vehicles Symposium (IV).

[43] Yang K., Hu X., Bergasa L.M., Romera E., Huang X., Sun D., Wang K. Can we PASS beyond the Field of View? Panoramic Annular Semantic Segmentation for Real-World Surrounding Perception. Proceedings of the 2019 IEEE Intelligent Vehicles Symposium (IV).

[44] Gallego G., Delbruck T., Orchard G.M., Bartolozzi C., Scaramuzza D. (2020). Event-based Vision: A Survey. IEEE Trans. Pattern Anal. Mach. Intell..

[45] Rebecq H., Horstschaefer T., Gallego G., Scaramuzza D. (2017). EVO: A Geometric Approach to Event-Based 6-DOF Parallel Tracking and Mapping in Real Time. IEEE Robot. Autom. Lett..

[46] Maqueda A., Loquercio A., Gallego G., García N., Scaramuzza D. Event-Based Vision Meets Deep Learning on Steering Prediction for Self-Driving Cars. Proceedings of the 2018 IEEE/CVF Conference on Computer Vision and Pattern Recognition.

[47] Lagorce X., Meyer C., Ieng S., Filliat D., Benosman R. (2015). Asynchronous Event-Based Multikernel Algorithm for High-Speed Visual Features Tracking. IEEE Trans. Neural Netw. Learn. Syst..

[48] Janai J., Güney F., Behl A., Geiger A. (2020). Computer vision for autonomous vehicles: Problems, datasets and state of the art. Found. Trends^®^ Comput. Graph. Vis..

[49] Wang Z., Lin L., Li Y. Multi-feature fusion based region of interest generation method for far-infrared pedestrian detection system. Proceedings of the 2018 IEEE Intelligent Vehicles Symposium (IV).

[50] Lee Y., Chan Y., Fu L., Hsiao P. (2015). Near-Infrared-Based Nighttime Pedestrian Detection Using Grouped Part Models. IEEE Trans. Intell. Transp. Syst..

[51] Mita S., Yuquan X., Ishimaru K., Nishino S. Robust 3D Perception for any Environment and any Weather Condition using Thermal Stereo. Proceedings of the 2019 IEEE Intelligent Vehicles Symposium (IV).

[52] Geiger A., Lenz P., Urtasun R. (2012). Are we ready for autonomous driving? The kitti vision benchmark suite. Proceedings of the 2012 IEEE Conference on Computer Vision and Pattern Recognition.

[53] Zheng Z., Wang B. (2012). On-Road Vehicle Detection based on Color Segmentation and Tracking Using Harris-SIFT. Adv. Mater. Res..

[54] Chen H.-T., Wu Y.-C., Hsu C.-C. (2015). Daytime preceding vehicle brake light detection using monocular vision. IEEE Sens. J..

[55] Zhang Y., Song B., Du X., Guizani M. (2018). Vehicle Tracking Using Surveillance with Multimodal Data Fusion. IEEE Trans. Intell. Transp. Syst..

[56] Swamy N., Srilekha S. Vehicle detection and counting based on color space model. Proceedings of the 2015 International Conference on Communications and Signal Processing (ICCSP).

[57] Anandhalli M., Baligar V. Vehicle Detection and Tracking Based on Color Feature. Proceedings of the 2017 International Conference on Recent Advances in Electronics and Communication Technology (ICRAECT).

[58] Razalli H., Ramli R., Alkawaz M.H. Emergency Vehicle Recognition and Classification Method Using HSV Color Segmentation. Proceedings of the 2020 16th IEEE International Colloquium on Signal Processing & Its Applications (CSPA).

[59] Song G.Y., Lee K.Y., Lee J.W. Vehicle detection by edge-based candidate generation and appearance-based classification. Proceedings of the 2008 IEEE Intelligent Vehicles Symposium.

[60] Jie T., Jian L., Xiangjing A. Learning proposal for front-vehicle detection. Proceedings of the 2015 Chinese Automation Congress (CAC).

[61] Nur S.A., Ibrahim M.M., Ali N.M., Nur F.I.Y. Vehicle detection based on underneath vehicle shadow using edge features. Proceedings of the 2016 6th IEEE International Conference on Control System, Computing and Engineering (ICCSCE).

[62] Jeong S., Kang S., Kim J. (2013). Vehicle Detection Based on the Use of Shadow Region and Edge. Proc. SPIE.

[63] Manana M., Tu C., Owolawi P.A. Preprocessed Faster RCNN for Vehicle Detection. Proceedings of the 2018 International Conference on Intelligent and Innovative Computing Applications (ICONIC).

[64] Rayavel P., Rathnavel P., Bharathi M., Kumar T.S. Dynamic Traffic Control System Using Edge Detection Algorithm. Proceedings of the 2018 International Conference on Soft-computing and Network Security (ICSNS).

[65] Aarthi R., Padmavathi S., Amudha J. Vehicle Detection in Static Images Using Color and Corner Map. Proceedings of the 2010 International Conference on Recent Trends in Information, Telecommunication and Computing.

[66] Munajat M.D.E., Widyantoro D.H., Munir R. Vehicle detection and tracking based on corner and lines adjacent detection features. Proceedings of the 2016 2nd International Conference on Science in Information Technology (ICSITech).

[67] Teoh S.S., Bräunl T. (2012). Symmetry-based monocular vehicle detection system. Mach. Vis. Appl..

[68] Jheng Y., Yen Y., Sun T. A symmetry-based forward vehicle detection and collision warning system on Android smartphone. Proceedings of the 2015 IEEE International Conference on Consumer Electronics.

[69] Zebbara K., Ansari M.E., Mazoul A., Oudani H. A Fast Road Obstacle Detection Using Association and Symmetry recognition. Proceedings of the 2019 International Conference on Wireless Technologies, Embedded and Intelligent Systems (WITS).

[70] Satzoda R.K., Trivedi M.M. (2016). Multipart Vehicle Detection Using Symmetry-Derived Analysis and Active Learning. IEEE Trans. Intell. Transp. Syst..

[71] Kalinke T., Tzomakas C., von Seelen W. A texture-based object detection and an adaptive model-based classification. Proceedings of the Procs. IEEE Intelligent Vehicles Symposium.

[72] Lin P., Xu J., Bian J. Robust Vehicle Detection in Vision Systems Based on Fast Wavelet Transform and Texture Analysis. Proceedings of the 2007 IEEE International Conference on Automation and Logistics.

[73] Qian Z., Shi H. Video Vehicle Detection Based on Texture Analysis. Proceedings of the 2010 Chinese Conference on Pattern Recognition (CCPR).

[74] Cheon M., Lee W., Yoon C., Park M. (2012). Vision-Based Vehicle Detection System with Consideration of the Detecting Location. IEEE Trans. Intell. Transp. Syst..

[75] He Y., Li J., Wang H., Pu H., Li R. Adaptive Vehicle Shadow Detection Algorithm in Highway. Proceedings of the 2012 Fifth International Symposium on Computational Intelligence and Design.

[76] Xia X., Lu X., Cao Y., Xia S., Fu C. (2019). Moving Vehicle Detection with Shadow Elimination Based on Improved ViBe Algorithm. J. Phys. Conf. Ser..

[77] Ibarra-Arenado M., Tjahjadi T., Pérez-Oria J., Robla-Gómez S., Jiménez-Avello A. (2017). Shadow-based vehicle detection in urban traffic. (in en). Sensors.

[78] López A., Hilgenstock J., Busse A., Baldrich R., Lumbreras F., Serrat J. Temporal coherence analysis for intelligent headlight control. Proceedings of the 2nd Workshop on Planning, Perception and Navigation for Intelligent Vehicles.

[79] Guo J., Wang J., Guo X., Yu C., Sun X.J.S. (2014). Preceding vehicle detection and tracking adaptive to illumination variation in night traffic scenes based on relevance analysis. Sensors.

[80] Kosaka N., Ohashi G. (2015). Vision-Based Nighttime Vehicle Detection Using CenSurE and SVM. IEEE Trans. Intell. Transp. Syst..

[81] Satzoda R.K., Trivedi M.M. (2016). Looking at Vehicles in the Night: Detection and Dynamics of Rear Lights. IEEE Trans. Intell. Transp. Syst..

[82] Zou Q., Ling H., Luo S., Huang Y., Tian M. (2015). Robust Nighttime Vehicle Detection by Tracking and Grouping Headlights. IEEE Trans. Intell. Transp. Syst..

[83] Kavya T.S., Tsogtbaatar E., Jang Y., Cho S. Night-time Vehicle Detection Based on Brake/Tail Light Color. Proceedings of the 2018 International SoC Design Conference (ISOCC).

[84] Kajabad E.N. Detection of Vehicle and Brake Light Based on Cascade and HSV Algorithm in Autonomous Vehicle. Proceedings of the 2018 International Conference on Industrial Engineering, Applications and Manufacturing (ICIEAM).

[85] Li Y., Er M.J., Shen D. (2015). A Novel Approach for Vehicle Detection Using an AND–OR-Graph-Based Multiscale Model. IEEE Trans. Intell. Transp. Syst..

[86] Rezaei M., Terauchi M., Klette R. (2015). Robust Vehicle Detection and Distance Estimation Under Challenging Lighting Conditions. IEEE Trans. Intell. Transp. Syst..

[87] Bi Q., Yang M., Wang C., Wang B. An Efficient Hierarchical Convolutional Neural Network for Traffic Object Detection. Proceedings of the 2018 IEEE Intelligent Vehicles Symposium (IV).

[88] Huang J., Hu H., Liu X., Liu L. Research on recognition of motional vehicle based on second-difference algorithm. Proceedings of the 2009 IEEE International Symposium on Industrial Electronics.

[89] Chen C., Zhang X. (2012). Moving Vehicle Detection Based on Union of Three-Frame Difference. Advances in Electronic Engineering, Communication and Management.

[90] Li W., Yao J., Dong T., Li H., He X. Moving vehicle detection based on an improved interframe difference and a Gaussian model. Proceedings of the 2015 8th International Congress on Image and Signal Processing (CISP).

[91] Congsheng L., Zhaoyang H. The System Design for Improving Vechicle Detection Precision Based on Image Processing. Proceedings of the 2016 International Conference on Smart Grid and Electrical Automation (ICSGEA).

[92] Ji W., Tang L., Li D., Yang W., Liao Q. (2016). Video-based construction vehicles detection and its application in intelligent monitoring system. Caai Trans. Intell. Technol..

[93] Stauffer C., Grimson W.E.L. (1999). Adaptive background mixture models for real-time tracking. Proceedings of the 1999 IEEE Computer Society Conference on Computer Vision and Pattern Recognition (Cat. No PR00149).

[94] KaewTraKulPong P., Bowden R., Remagnino P., Jones G.A., Paragios N., Regazzoni C.S. (2002). An Improved Adaptive Background Mixture Model for Real-time Tracking with Shadow Detection. Video-Based Surveillance Systems: Computer Vision and Distributed Processing.

[95] Kim K., Chalidabhongse T.H., Harwood D., Davis L. (2005). Real-time foreground–background segmentation using codebook model. Real-Time Imaging.

[96] Ilyas A., Scuturici M., Miguet S. Real Time Foreground-Background Segmentation Using a Modified Codebook Model. Proceedings of the 2009 Sixth IEEE International Conference on Advanced Video and Signal Based Surveillance.

[97] Barnich O., Droogenbroeck M.V. (2011). ViBe: A Universal Background Subtraction Algorithm for Video Sequences. IEEE Trans. Image Process..

[98] Pan C., Zhu Z., Jiang L., Wang M., Lu X. Adaptive ViBe background model for vehicle detection. Proceedings of the 2017 IEEE 2nd Advanced Information Technology, Electronic and Automation Control Conference (IAEAC).

[99] Charouh Z., Ghogho M., Guennoun Z. Improved Background Subtraction-based Moving Vehicle Detection by Optimizing Morphological Operations using Machine Learning. Proceedings of the 2019 IEEE International Symposium on INnovations in Intelligent SysTems and Applications (INISTA).

[100] Meinhardt-Llopis E., Pérez J.S., Kondermann D. (2013). Horn-Schunck Optical Flow with a Multi-Scale Strategy. Image Process. Line.

[101] Bruhn A., Weickert J., Schnörr C. (2005). Lucas/Kanade meets Horn/Schunck: Combining local and global optic flow methods. Int. J. Comput. Vis..

[102] Chen Y., Wu Q. Moving vehicle detection based on optical flow estimation of edge. Proceedings of the 2015 11th International Conference on Natural Computation (ICNC).

[103] Guo Z., Zhou Z., Sun X. Vehicle detection and tracking based on optical field. Proceedings of the 2017 International Conference on Security, Pattern Analysis, and Cybernetics (SPAC).

[104] Gomaa A., Abdelwahab M.M., Abo-Zahhad M., Minematsu T., Taniguchi R.-I. (2019). Robust Vehicle Detection and Counting Algorithm Employing a Convolution Neural Network and Optical Flow. Sensors.

[105] Bertozzi M., Broggi A. (1998). GOLD: A parallel real-time stereo vision system for generic obstacle and lane detection. IEEE Trans. Image Process..

[106] Knoeppel C., Schanz A., Michaelis B. (2000). Robust vehicle detection at large distance using low resolution cameras. Proceedings of the IEEE Intelligent Vehicles Symposium 2000 (Cat. No. 00TH8511).

[107] Bertozzi M., Bombini L., Cerri P., Medici P., Antonello P.C., Miglietta M. (2008). Obstacle detection and classification fusing radar and vision. Proceedings of the 2008 IEEE Intelligent Vehicles Symposium.

[108] Lin Y., Lin C., Chen L., Chen C. Adaptive IPM-based lane filtering for night forward vehicle detection. Proceedings of the 2011 6th IEEE Conference on Industrial Electronics and Applications.

[109] Li L., Wang B., Wang H., Zhang J., Luan Y., Wang W., Guo R. Road edge and obstacle detection on the SmartGuard navigation system. Proceedings of the 2014 3rd International Conference on Applied Robotics for the Power Industry.

[110] Wongsaree P., Sinchai S., Wardkein P., Koseeyaporn J. Distance Detection Technique Using Enhancing Inverse Perspective Mapping. Proceedings of the 2018 3rd International Conference on Computer and Communication Systems (ICCCS).

[111] Kim Y., Kum D. Deep Learning based Vehicle Position and Orientation Estimation via Inverse Perspective Mapping Image. Proceedings of the 2019 IEEE Intelligent Vehicles Symposium (IV).

[112] Labayrade R., Aubert D., Tarel J.-P. (2002). Real time obstacle detection in stereovision on non flat road geometry through “v-disparity” representation. Proceedings of the Intelligent Vehicle Symposium, 2002. IEEE.

[113] Hu Z., Uchimura K. (2005). UV-disparity: An efficient algorithm for stereovision based scene analysis. Proceedings of the IEEE Proceedings. Intelligent Vehicles Symposium, 2005.

[114] Nguyen V.D., Nguyen T.T., Nguyen D.D., Jeon J.W. Toward Real-Time Vehicle Detection Using Stereo Vision and an Evolutionary Algorithm. Proceedings of the 2012 IEEE 75th Vehicular Technology Conference (VTC Spring).

[115] Cai Y., Chen X., Wang H., Chen L. Deep representation and stereo vision based vehicle detection. Proceedings of the 2015 IEEE International Conference on Cyber Technology in Automation, Control, and Intelligent Systems (CYBER).

[116] Dekkiche D., Vincke B., Mérigot A. (2015). Vehicles Detection in Stereo Vision Based on Disparity Map Segmentation and Objects Classification. Proceedings of the International Symposium on Visual Computing.

[117] Coenen M., Rottensteiner F., Heipke C. (2017). Detection and 3d modelling of vehicles from terrestrial stereo image pairs. Int. Arch. Photogramm. Remote Sens. Spat. Inf. Sci..

[118] Wang J., Chen S.J., Zhou L., Wan K., Yau W. Vehicle Detection and Width Estimation in Rain by Fusing Radar and Vision. Proceedings of the 2018 15th International Conference on Control, Automation, Robotics and Vision (ICARCV).

[119] Leng J., Liu Y., Du D., Zhang T., Quan P. (2019). Robust Obstacle Detection and Recognition for Driver Assistance Systems. IEEE Trans. Intell. Transp. Syst..

[120] Lefebvre S., Ambellouis S. Vehicle detection and tracking using Mean Shift segmentation on semi-dense disparity maps. Proceedings of the 2012 IEEE Intelligent Vehicles Symposium.

[121] Park J., Yoon J.H., Park M., Yoon K. (2014). Dynamic Point Clustering with Line Constraints for Moving Object Detection in DAS. IEEE Signal Process. Lett..

[122] Ridel D.A., Shinzato P.Y., Wolf D.F. A Clustering-Based Obstacle Segmentation Approach for Urban Environments. Proceedings of the 2015 12th Latin American Robotics Symposium and 2015 3rd Brazilian Symposium on Robotics (LARS-SBR).

[123] Chen L., Fan L., Xie G., Huang K., Nüchter A. (2017). Moving-Object Detection from Consecutive Stereo Pairs Using Slanted Plane Smoothing. IEEE Trans. Intell. Transp. Syst..

[124] Königshof H., Salscheider N.O., Stiller C. Realtime 3D Object Detection for Automated Driving Using Stereo Vision and Semantic Information. Proceedings of the 2019 IEEE Intelligent Transportation Systems Conference (ITSC).

[125] Geiger A., Kitt B. Object flow: A descriptor for classifying traffic motion. Proceedings of the 2010 IEEE Intelligent Vehicles Symposium.

[126] Kim G., Cho J. Vision-based vehicle detection and inter-vehicle distance estimation. Proceedings of the 2012 12th International Conference on Control, Automation and Systems.

[127] Min Q., Huang Y. (2016). Moving object detection based on combined stereovision and optical flow. Opt. Tech..

[128] Jun W., Tao M., Bin K., Hu W. An approach of lane detection based on Inverse Perspective Mapping. Proceedings of the 17th International IEEE Conference on Intelligent Transportation Systems (ITSC).

[129] Ozgunalp U., Dahnoun N. Lane detection based on improved feature map and efficient region of interest extraction. Proceedings of the 2015 IEEE Global Conference on Signal and Information Processing (GlobalSIP).

[130] Ying Z., Li G. Robust lane marking detection using boundary-based inverse perspective mapping. Proceedings of the 2016 IEEE International Conference on Acoustics, Speech and Signal Processing (ICASSP).

[131] Hamzah R.A., Ibrahim H. (2016). Literature survey on stereo vision disparity map algorithms. J. Sens..

[132] Parodi P., Piccioli G. A feature-based recognition scheme for traffic scenes. Proceedings of the Intelligent Vehicles ’95. Symposium.

[133] Handmann U., Kalinke T., Tzomakas C., Werner M., Seelen W.v. (2000). An image processing system for driver assistance. Image Vis. Comput..

[134] Bensrhair A., Bertozzi M., Broggi A., Miche P., Mousset S., Toulminet G. (2001). A cooperative approach to vision-based vehicle detection. ITSC 2001. 2001 IEEE Intelligent Transportation Systems. Proceedings (Cat. No.01TH8585).

[135] Li Y., Li B., Tian B., Zhu F., Xiong G., Wang K. Vehicle detection based on the deformable hybrid image template. Proceedings of the 2013 IEEE International Conference on Vehicular Electronics and Safety.

[136] Wang J., Zhang S., Chen J. Vehicle Detection by Sparse Deformable Template Models. Proceedings of the 2014 IEEE 17th International Conference on Computational Science and Engineering.

[137] Li D.L., Prasad M., Liu C., Lin C. (2020). Multi-View Vehicle Detection Based on Fusion Part Model with Active Learning. IEEE Trans. Intell. Transp. Syst..

[138] Dalal N., Triggs B. (2005). Histograms of oriented gradients for human detection. Proceedings of the 2005 IEEE Computer Society Conference on Computer Vision and Pattern Recognition (CVPR’05).

[139] Li X., Guo X. (2013). A HOG Feature and SVM Based Method for Forward Vehicle Detection with Single Camera. Proceedings of the 2013 5th International Conference on Intelligent Human-Machine Systems and Cybernetics.

[140] Arróspide J., Salgado L., Camplani M. (2013). Image-based on-road vehicle detection using cost-effective Histograms of Oriented Gradients. J. Vis. Commun. Image Represent..

[141] Laopracha N., Sunat K. (2017). Comparative Study of Computational Time that HOG-Based Features Used for Vehicle Detection. Proceedings of the International Conference on Computing and Information Technology.

[142] Laopracha N., Thongkrau T., Sunat K., Songrum P., Chamchong R. Improving vehicle detection by adapting parameters of HOG and kernel functions of SVM. Proceedings of the 2014 International Computer Science and Engineering Conference (ICSEC).

[143] Lee S.H., Bang M., Jung K., Yi K. An efficient selection of HOG feature for SVM classification of vehicle. Proceedings of the 2015 International Symposium on Consumer Electronics (ISCE).

[144] Zakaria Y., el Munim H.E.A., Ghoneima M., Hammad S. (2018). Modified HOG based on-road vehicle detection method. Int. J. Pure Appl. Math..

[145] Naiel M.A., Ahmad M.O., Swamy M.N.S. (2019). A vehicle detection scheme based on two-dimensional HOG features in the DFT and DCT domains. Multidimens. Syst. Signal Process..

[146] Sun Z., Bebis G., Miller R. (2005). On-road vehicle detection using evolutionary Gabor filter optimization. IEEE Trans. Intell. Transp. Syst..

[147] Arróspide J., Salgado L. (2013). Log-Gabor Filters for Image-Based Vehicle Verification. IEEE Trans. Image Process..

[148] David H., Athira T.A. Improving the Performance of Vehicle Detection and Verification by Log Gabor Filter Optimization. Proceedings of the 2014 Fourth International Conference on Advances in Computing and Communications.

[149] Field D. (1987). Relations between the statistics of natural images and the response properties of cortical cells. Josa A.

[150] Zhang R.-H., You F., Chen F., He W.-Q. (2018). Vehicle detection method for intelligent vehicle at night time based on video and laser information. Int. J. Pattern Recognit. Artif. Intell..

[151] Truong Q.B., Lee B.R. (2009). Vehicle detection algorithm using hypothesis generation and verification. Proceedings of the International Conference on Intelligent Computing.

[152] Suppatoomsin C., Srikaew A. (2011). 2DPCA for vehicle detection from CCTV captured image. Proceedings of the 2011 International Conference on Information Science and Applications.

[153] Chompoo S., Arthit S. (2013). Hybrid Method for Vehicle Detection from CCTV captured image. Advanced Materials Research.

[154] Wu C.-C., Weng K.-W. (2017). The detecting and tracking system for vehicles. Proceedings of the 2017 10th International Conference on Ubi-media Computing and Workshops (Ubi-Media).

[155] Viola P., Jones M. (2001). Rapid object detection using a boosted cascade of simple features. Proceedings of the 2001 IEEE Computer Society Conference on Computer Vision and Pattern Recognition. CVPR 2001.

[156] Haselhoff A., Kummert A. (2009). A vehicle detection system based on haar and triangle features. In Proceedings of the 2009 IEEE Intelligent Vehicles Symposium.

[157] Qiu Q.-J., Liu Y., Cai D.-W. Vehicle detection based on LBP features of the Haar-like Characteristics. In Proceeding of the 11th World Congress on Intelligent Control and Automation.

[158] Jabri S., Saidallah M., El Belrhiti El Alaoui A., Fergougui A.E.L. Moving Vehicle Detection Using Haar-like, LBP and a Machine Learning Adaboost Algorithm. Proceedings of the 2018 IEEE International Conference on Image Processing, Applications and Systems (IPAS).

[159] Chen D., Jin G., Lu L., Tan L., Wei W. Infrared Image Vehicle Detection Based on Haar-like Feature. Proceedings of the 2018 IEEE 3rd Advanced Information Technology, Electronic and Automation Control Conference (IAEAC).

[160] Lowe G. (2004). Distinctive image features from scale-invariant keypoints. Int. J. Comput. Vis..

[161] Chen X., Meng Q. Vehicle Detection from UAVs by Using SIFT with Implicit Shape Model. Proceedings of the 2013 IEEE International Conference on Systems, Man, and Cybernetics.

[162] Cai Y., Li L., Ni S., Lv J., Zeng W., Yuanlong Y. Moving vehicle detection based on dense SIFT and Extreme Learning Machine for visual surveillance. Proceedings of the 2015 IEEE International Conference on Robotics and Biomimetics (ROBIO).

[163] Xu Y., Zhang J., Liu C., Gu J., Hua L. Vehicle Recognition Method Based on Color Invariant SIFT Features. Proceedings of the 2018 37th Chinese Control Conference (CCC).

[164] Momin B.F., Kumbhare S.M. Vehicle detection in video surveillance system using Symmetrical SURF. Proceedings of the 2015 IEEE International Conference on Electrical, Computer and Communication Technologies (ICECCT).

[165] Shrivastava A., Arulmohzivarman P. Vehicle direction detection using symmetrical SURF and centroid point calculation. Proceedings of the 2016 International Conference on Communication and Signal Processing (ICCSP).

[166] Shujuan S., Zhize X., Xingang W., Guan H., Wenqi W., De X. Real-time vehicle detection using Haar-SURF mixed features and gentle AdaBoost classifier. Proceedings of the 27th Chinese Control and Decision Conference (2015 CCDC).

[167] Sajib M.S.R., Tareeq S.M. A feature based method for real time vehicle detection and classification from on-road videos. Proceedings of the 2017 20th International Conference of Computer and Information Technology (ICCIT).

[168] Krizhevsky A., Sutskever I., Hinton G. (2012). ImageNet Classification with Deep Convolutional Neural Networks. Neural Inf. Process. Syst..

[169] Girshick R., Donahue J., Darrell T., Malik J. Rich feature hierarchies for accurate object detection and semantic segmentation. Proceedings of the IEEE Conference on Computer Vision and Pattern Recognition.

[170] He K., Zhang X., Ren S., Sun J. (2014). Spatial Pyramid Pooling in Deep Convolutional Networks for Visual Recognition. Computer Vision—ECCV 2014.

[171] Girshick R. Fast R-CNN. Proceedings of the 2015 IEEE International Conference on Computer Vision (ICCV).

[172] Ren S., He K., Girshick R., Sun J. (2017). Faster R-CNN: Towards Real-Time Object Detection with Region Proposal Networks. IEEE Trans. Pattern Anal. Mach. Intell..

[173] Girshick R., Donahue J., Darrell T., Malik J. (2016). Region-Based Convolutional Networks for Accurate Object Detection and Segmentation. IEEE Trans. Pattern Anal. Mach. Intell..

[174] Baek I., Davies A., Yan G., Rajkumar R.R. Real-time Detection, Tracking, and Classification of Moving and Stationary Objects using Multiple Fisheye Images. Proceedings of the 2018 IEEE Intelligent Vehicles Symposium (IV).

[175] Gündüz G., Acarman T. A Lightweight Online Multiple Object Vehicle Tracking Method. Proceedings of the 2018 IEEE Intelligent Vehicles Symposium (IV).

[176] Zhang H., Wang K., Tian Y., Gou C., Wang F. (2018). MFR-CNN: Incorporating Multi-Scale Features and Global Information for Traffic Object Detection. IEEE Trans. Veh. Technol..

[177] Hu X., Xu X., Xiao Y., Chen H., He S., Qin J., Heng P.-A. (2019). SINet: A Scale-Insensitive Convolutional Neural Network for Fast Vehicle Detection. IEEE Trans. Intell. Transp. Syst..

[178] Lee K.H., Tagawa T., Pan J.M., Gaidon A., Douillard B. An Attention-based Recurrent Convolutional Network for Vehicle Taillight Recognition. Proceedings of the 2019 IEEE Intelligent Vehicles Symposium (IV).

[179] Shin K., Kwon Y.P., Tomizuka M. RoarNet: A Robust 3D Object Detection based on RegiOn Approximation Refinement. Proceedings of the 2019 IEEE Intelligent Vehicles Symposium (IV).

[180] Redmon J., Divvala S., Girshick R., Farhadi A. You only look once: Unified, real-time object detection. Proceedings of the IEEE Conference on Computer Vision and Pattern Recognition.

[181] Liu W., Anguelov D., Erhan D., Szegedy C., Reed S., Fu C., Berg A.C. (2016). Ssd: Single shot multibox detector. Proceedings of the European Conference on Computer Vision.

[182] Redmon J., Farhadi A. YOLO9000: Better, Faster, Stronger. Proceedings of the IEEE Conference on Computer Vision and Pattern Recognition.

[183] Redmon J., Farhadi A. (2018). Yolov3: An incremental improvement. arXiv.

[184] Bochkovskiy A., Wang C.-Y., Liao H.-Y.M. (2020). YOLOv4: Optimal Speed and Accuracy of Object Detection. arXiv.

[185] YOLOv5. https://github.com/ultralytics/yolov5.

[186] Gählert N., Mayer M., Schneider L., Franke U., Denzler J. MB-Net: MergeBoxes for Real-Time 3D Vehicles Detection. Proceedings of the 2018 IEEE Intelligent Vehicles Symposium (IV).

[187] Chen L., Zou Q., Pan Z., Lai D., Zhu L., Hou Z., Wang J., Cao D. (2019). Surrounding Vehicle Detection Using an FPGA Panoramic Camera and Deep CNNs. IEEE Trans. Intell. Transp. Syst..

[188] Gählert N., Wan J., Weber M., Zöllner J.M., Franke U., Denzler J. Beyond Bounding Boxes: Using Bounding Shapes for Real-Time 3D Vehicle Detection from Monocular RGB Images. Proceedings of the 2019 IEEE Intelligent Vehicles Symposium (IV).

[189] Jiang Q., Zhang L., Meng D. Target Detection Algorithm Based on MMW Radar and Camera Fusion*. Proceedings of the 2019 IEEE Intelligent Transportation Systems Conference (ITSC).

[190] Cheng Z., Wang Z., Huang H., Liu Y. Dense-ACSSD for End-to-end Traffic Scenes Recognition. Proceedings of the 2019 IEEE Intelligent Vehicles Symposium (IV).

[191] Lin C.T., Chen S., Santoso P.S., Lin H., Lai S. (2020). Real-Time Single-Stage Vehicle Detector Optimized by Multi-Stage Image-Based Online Hard Example Mining. IEEE Trans. Veh. Technol..

[192] Kiela K., Barzdenas V., Jurgo M., Macaitis V., Rafanavicius J., Vasjanov A., Kladovscikov L., Navickas R. (2020). Review of V2X–IoT Standards and Frameworks for ITS Applications. Appl. Sci..

[193] Azim A., Aycard O. Detection, classification and tracking of moving objects in a 3D environment. Proceedings of the 2012 IEEE Intelligent Vehicles Symposium.

[194] Christina G., Gustav T., Tomas C., Håkan L. (2011). Spatial filtering for detection of partly occluded targets. Opt. Eng..

[195] Yi Y., Guang Y., Hao Z., Meng-yin F., Mei-ling W. Moving object detection under dynamic background in 3D range data. Proceedings of the 2014 IEEE Intelligent Vehicles Symposium Proceedings.

[196] Naujoks B., Burger P., Wuensche H. Fast 3D Extended Target Tracking using NURBS Surfaces. Proceedings of the 2019 IEEE Intelligent Transportation Systems Conference (ITSC).

[197] Ye E., Althoff M. Model-based Offline Vehicle Tracking in Automotive Applications Using a Precise 3D Model. Proceedings of the 2019 IEEE Intelligent Transportation Systems Conference (ITSC).

[198] Petrovskaya A., Thrun S. (2009). Model based vehicle detection and tracking for autonomous urban driving. Auton. Robot..

[199] Baur S.A., Moosmann F., Wirges S., Rist C.B. Real-time 3D LiDAR Flow for Autonomous Vehicles. Proceedings of the 2019 IEEE Intelligent Vehicles Symposium (IV).

[200] Liu K., Wang W., Tharmarasa R., Wang J. (2019). Dynamic Vehicle Detection with Sparse Point Clouds Based on PE-CPD. IEEE Trans. Intell. Transp. Syst..

[201] Wu B., Wan A., Yue X., Keutzer K. (2018). Squeezeseg: Convolutional neural nets with recurrent crf for real-time road-object segmentation from 3d lidar point cloud. Proceedings of the 2018 IEEE International Conference on Robotics and Automation (ICRA).

[202] Wang Y., Shi T., Yun P., Tai L., Liu M. (2018). Pointseg: Real-time semantic segmentation based on 3d lidar point cloud. arXiv.

[203] Premebida C., Garrote L., Asvadi A., Ribeiro A.P., Nunes U. High-resolution LIDAR-based depth mapping using bilateral filter. Proceedings of the 2016 IEEE 19th International Conference on Intelligent Transportation Systems (ITSC).

[204] Li B., Zhang T., Xia T. (2016). Vehicle Detection from 3D Lidar Using Fully Convolutional Network. arXiv.

[205] Asvadi A., Garrote L., Premebida C., Peixoto P., Nunes U.J. (2017). Depthcn: Vehicle detection using 3d-lidar and convnet. Proceedings of the 2017 IEEE 20th International Conference on Intelligent Transportation Systems (ITSC).

[206] Asvadi A., Garrote L., Premebida C., Peixoto P., Nunes U.J. (2018). Real-Time Deep ConvNet-Based Vehicle Detection Using 3D-LIDAR Reflection Intensity Data. Proceedings of the ROBOT 2017: Third Iberian Robotics Conference.

[207] Banerjee K., Notz D., Windelen J., Gavarraju S., He M. Online Camera LiDAR Fusion and Object Detection on Hybrid Data for Autonomous Driving. Proceedings of the 2018 IEEE Intelligent Vehicles Symposium (IV).

[208] Feng D., Wei X., Rosenbaum L., Maki A., Dietmayer K. Deep Active Learning for Efficient Training of a LiDAR 3D Object Detector. Proceedings of the 2019 IEEE Intelligent Vehicles Symposium (IV).

[209] Chen X., Ma H., Wan J., Li B., Xia T. Multi-view 3d object detection network for autonomous driving. Proceedings of the IEEE Conference on Computer Vision and Pattern Recognition.

[210] Beltrán J., Guindel C., Moreno F.M., Cruzado D., Garcia F., de la Escalera A. (2018). Birdnet: A 3d object detection framework from lidar information. Proceedings of the 2018 21st International Conference on Intelligent Transportation Systems (ITSC).

[211] Feng D., Rosenbaum L., Dietmayer K. (2018). Towards safe autonomous driving: Capture uncertainty in the deep neural network for lidar 3d vehicle detection. Proceedings of the 2018 21st International Conference on Intelligent Transportation Systems (ITSC).

[212] Simon M., Milz S., Amende K., Gross H. (2018). Complex-YOLO: Real-time 3D Object Detection on Point Clouds. arXiv.

[213] Yu S.-L., Westfechtel T., Hamada R., Ohno K., Tadokoro S. (2017). Vehicle detection and localization on bird’s eye view elevation images using convolutional neural network. Proceedings of the 2017 IEEE International Symposium on Safety, Security and Rescue Robotics (SSRR).

[214] Ali W., Abdelkarim S., Zidan M., Zahran M., el Sallab A. Yolo3d: End-to-end real-time 3d oriented object bounding box detection from lidar point cloud. Proceedings of the European Conference on Computer Vision (ECCV).

[215] Wang Z., Zhan W., Tomizuka M. Fusing Bird’s Eye View LIDAR Point Cloud and Front View Camera Image for 3D Object Detection. Proceedings of the 2018 IEEE Intelligent Vehicles Symposium (IV).

[216] Yang B., Luo W., Urtasun R. Pixor: Real-time 3d object detection from point clouds. Proceedings of the IEEE conference on Computer Vision and Pattern Recognition.

[217] Feng D., Rosenbaum L., Timm F., Dietmayer K. Leveraging Heteroscedastic Aleatoric Uncertainties for Robust Real-Time LiDAR 3D Object Detection. Proceedings of the 2019 IEEE Intelligent Vehicles Symposium (IV).

[218] Kim S., Kim H., Yoo W., Huh K. (2016). Sensor Fusion Algorithm Design in Detecting Vehicles Using Laser Scanner and Stereo Vision. IEEE Trans. Intell. Transp. Syst..

[219] An J., Choi B., Kim H., Kim E. (2019). A New Contour-Based Approach to Moving Object Detection and Tracking Using a Low-End Three-Dimensional Laser Scanner. IEEE Trans. Veh. Technol..

[220] Engelcke M., Rao D., Wang D.Z., Tong C.H., Posner I. (2017). Vote3deep: Fast object detection in 3d point clouds using efficient convolutional neural networks. Proceedings of the 2017 IEEE International Conference on Robotics and Automation (ICRA).

[221] Li B. (2017). 3d fully convolutional network for vehicle detection in point cloud. Proceedings of the 2017 IEEE/RSJ International Conference on Intelligent Robots and Systems (IROS).

[222] Du X., Ang M.H., Karaman S., Rus D. (2018). A general pipeline for 3d detection of vehicles. Proceedings of the 2018 IEEE International Conference on Robotics and Automation (ICRA).

[223] Zhou Y., Tuzel O. Voxelnet: End-to-end learning for point cloud based 3d object detection. Proceedings of the IEEE Conference on Computer Vision and Pattern Recognition.

[224] Yan Y., Mao Y., Li B. (2018). Second: Sparsely embedded convolutional detection. Sensors.

[225] Shi S., Wang Z., Shi J., Wang X., Li H. (2020). From points to parts: 3d object detection from point cloud with part-aware and part-aggregation network. IEEE Trans. Pattern Anal. Mach. Intell..

[226] Sindagi V.A., Zhou Y., Tuzel O. (2019). MVX-Net: Multimodal voxelnet for 3D object detection. Proceedings of the 2019 International Conference on Robotics and Automation (ICRA).

[227] Morales N., Toledo J., Acosta L., Sánchez-Medina J. (2017). A Combined Voxel and Particle Filter-Based Approach for Fast Obstacle Detection and Tracking in Automotive Applications. IEEE Trans. Intell. Transp. Syst..

[228] Qi R., Su H., Mo K., Guibas L.J. Pointnet: Deep learning on point sets for 3d classification and segmentation. Proceedings of the IEEE Conference on Computer Vision and Pattern Recognition.

[229] Qi R., Yi L., Su H., Guibas L.J. (2017). Pointnet++: Deep hierarchical feature learning on point sets in a metric space. Advances in Neural Information Processing Systems.

[230] Li Y., Bu R., Sun M., Wu W., Di X., Chen B. Pointcnn: Convolution on x-transformed points. Proceedings of the Advances in Neural Information Processing Systems.

[231] Yang Z., Sun Y., Liu S., Shen X., Jia J. (2018). Ipod: Intensive point-based object detector for point cloud. arXiv.

[232] Lang H., Vora S., Caesar H., Zhou L., Yang J., Beijbom O. PointPillars: Fast encoders for object detection from point clouds. Proceedings of the IEEE Conference on Computer Vision and Pattern Recognition.

[233] Shi S., Wang X., Li H. Pointrcnn: 3d object proposal generation and detection from point cloud. Proceedings of the IEEE Conference on Computer Vision and Pattern Recognition.

[234] Han S., Wang X., Xu L., Sun H., Zheng N. (2016). Frontal object perception for Intelligent Vehicles based on radar and camera fusion. Proceedings of the 2016 35th Chinese Control Conference (CCC).

[235] Wang X., Xu L., Sun H., Xin J., Zheng N. (2016). On-Road Vehicle Detection and Tracking Using MMW Radar and Monovision Fusion. IEEE Trans. Intell. Transp. Syst..

[236] Wangyu Z., Bijun L.I., Yunxiao S., Haoda X.U. (2019). Vehicle Detection and Tracking Based on Fusion of Millimeter Wave Radar and Monocular Vision. Geomat. Inf. Sci. Wuhan Univ..

[237] Song W., Yang Y., Fu M., Qiu F., Wang M. (2017). Real-time obstacles detection and status classification for collision warning in a vehicle active safety system. IEEE Trans. Intell. Transp. Syst..

[238] Schumann O., Wöhler C., Hahn M., Dickmann J. Comparison of random forest and long short-term memory network performances in classification tasks using radar. Proceedings of the 2017 Sensor Data Fusion: Trends, Solutions, Applications (SDF).

[239] Scheiner N., Appenrodt N., Dickmann J., Sick B. Radar-based Feature Design and Multiclass Classification for Road User Recognition. Proceedings of the 2018 IEEE Intelligent Vehicles Symposium (IV).

[240] Akita T., Mita S. Object Tracking and Classification Using Millimeter-Wave Radar Based on LSTM. Proceedings of the 2019 IEEE Intelligent Transportation Systems Conference (ITSC).

[241] Scheiner N., Appenrodt N., Dickmann J., Sick B. Radar-based Road User Classification and Novelty Detection with Recurrent Neural Network Ensembles. Proceedings of the 2019 IEEE Intelligent Vehicles Symposium (IV).

[242] Schumann O., Hahn M., Dickmann J., Wöhler C. Semantic Segmentation on Radar Point Clouds. Proceedings of the 2018 21st International Conference on Information Fusion (FUSION).

[243] Danzer A., Griebel T., Bach M., Dietmayer K. 2D Car Detection in Radar Data with PointNets. Proceedings of the 2019 IEEE Intelligent Transportation Systems Conference (ITSC).

[244] Palffy A., Dong J., Kooij J.F.P., Gavrila D.M. (2020). CNN Based Road User Detection Using the 3D Radar Cube. IEEE Robot. Autom. Lett..

[245] Solimene R., Catapano I., Gennarelli G., Cuccaro A., Dell’Aversano A., Soldovieri F. (2014). SAR Imaging Algorithms and Some Unconventional Applications: A unified mathematical overview. IEEE Signal Process. Mag..

[246] Ma X., Wu P., Wu Y., Shen H. (2018). A Review on Recent Developments in Fully Polarimetric SAR Image Despeckling. IEEE J. Sel. Top. Appl. Earth Obs. Remote Sens..

[247] Bi H., Zhu D., Bi G., Zhang B., Hong W., Wu Y. (2020). FMCW SAR Sparse Imaging Based on Approximated Observation: An Overview on Current Technologies. IEEE J. Sel. Top. Appl. Earth Obs. Remote Sens..

[248] Yamada H., Kobayashi T., Yamaguchi Y., Sugiyama Y. High-resolution 2D SAR imaging by the millimeter-wave automobile radar. Proceedings of the 2017 IEEE Conference on Antenna Measurements & Applications (CAMA).

[249] Wang R., Pei J., Zhang Y., Li M., Huang Y., Wu J. An Auxiliary Parking Method Based on Automotive Millimeter wave SAR. Proceedings of the IGARSS 2019—2019 IEEE International Geoscience and Remote Sensing Symposium.

[250] Wang C., Pei J., Li M., Zhang Y., Huang Y., Yang J. Parking information perception based on automotive millimeter wave SAR. Proceedings of the 2019 IEEE Radar Conference (RadarConf).

[251] Gao X., Roy S., Xing G. (2021). MIMO-SAR: A Hierarchical High-resolution Imaging Algorithm for FMCW Automotive Radar. arXiv.

[252] Kim J., Hong S., Baek J., Kim E., Lee H. Autonomous vehicle detection system using visible and infrared camera. Proceedings of the 2012 12th International Conference on Control, Automation and Systems.

[253] Gu J., Xiao H., He W., Wang S., Wang X., Yuan K. FPGA based real-time vehicle detection system under complex background. Proceedings of the 2016 IEEE International Conference on Mechatronics and Automation.

[254] Dickson C.N., Wallace A.M., Kitchin M., Connor B. Improving infrared vehicle detection with polarisation. Proceedings of the IET Intelligent Signal Processing Conference 2013 (ISP 2013).

[255] Wen-jing C., Lu-ping W., Lu-ping Z. (2016). Vehicle detection algorithm based on SLPP-SHOG in infrared image. Laser Infrared.

[256] Nannan Q., Pengfei J., Li Y., Tan Y. (2017). Infrared vehicle detection based on visual saliency and target confidence. Infrared Laser Eng..

[257] Zhang X., Zhu X. Vehicle Detection in the Aerial Infrared Images via an Improved Yolov3 Network. Proceedings of the 2019 IEEE 4th International Conference on Signal and Image Processing (ICSIP).

[258] Xiaofeng Z., Mingyang X., Danpiao W., Jiaxing Y., Zhili Z. (2019). Infrared camouflage detection method for special vehicles based on improved SSD. Infrared Laser Eng..

[259] Wiesmann G., Schraml S., Litzenberger M., Belbachir A.N., Hofstätter M., Bartolozzi C. Event-driven embodied system for feature extraction and object recognition in robotic applications. Proceedings of the 2012 IEEE Computer Society Conference on Computer Vision and Pattern Recognition Workshops.

[260] Lagorce X., Orchard G., Galluppi F., Shi B.E., Benosman R.B. (2017). HOTS: A Hierarchy of Event-Based Time-Surfaces for Pattern Recognition. IEEE Trans. Pattern Anal. Mach. Intell..

[261] Sironi A., Brambilla M., Bourdis N., Lagorce X., Benosman R. HATS: Histograms of averaged time surfaces for robust event-based object classification. Proceedings of the IEEE Conference on Computer Vision and Pattern Recognition.

[262] Li J., Dong S., Yu Z., Tian Y., Huang T. Event-Based Vision Enhanced: A Joint Detection Framework in Autonomous Driving. Proceedings of the 2019 IEEE International Conference on Multimedia and Expo (ICME).

[263] Fayyad J., Jaradat M., Gruyer D., Najjaran H. (2020). Deep Learning Sensor Fusion for Autonomous Vehicle Perception and Localization: A Review. Sensors.

[264] Tsai C., Lai Y., Li Y., Guo J. A vision radar system for car safety driving applications. Proceedings of the 2017 International Symposium on VLSI Design, Automation and Test (VLSI-DAT).

[265] Perna S., Soldovieri F., Amin M. (2020). Editorial for Special Issue. Radar Imaging in Challenging Scenarios from Smart and Flexible Platforms. Remote Sens..

[266] Chavez-Garcia R.O., Aycard O. (2016). Multiple Sensor Fusion and Classification for Moving Object Detection and Tracking. IEEE Trans. Intell. Transp. Syst..

[267] Feng Q., Qi S., Li J., Dai B. Radar-Vision Fusion for Correcting the Position of Target Vehicles. Proceedings of the 2018 10th International Conference on Intelligent Human-Machine Systems and Cybernetics (IHMSC).

[268] Garcia F., Martin D., Escalera A.d.l., Armingol J.M. (2017). Sensor Fusion Methodology for Vehicle Detection. IEEE Intell. Transp. Syst. Mag..

[269] Deng J., Czarnecki K. MLOD: A multi-view 3D object detection based on robust feature fusion method. Proceedings of the 2019 IEEE Intelligent Transportation Systems Conference (ITSC).

[270] Aijazi A.K., Checchin P., Trassoudaine L. Multi sensorial data fusion for efficient detection and tracking of road obstacles for inter-distance and anti-colision safety management. Proceedings of the 2017 3rd International Conference on Control, Automation and Robotics (ICCAR).

[271] Koenig N., Howard A. (2004). Design and use paradigms for gazebo, an open-source multi-robot simulator. Proceedings of the 2004 IEEE/RSJ International Conference on Intelligent Robots and Systems (IROS) (IEEE Cat. No. 04CH37566).

[272] Kato S., Takeuchi E., Ishiguro Y., Ninomiya Y., Takeda K., Hamada T. (2015). An open approach to autonomous vehicles. IEEE Micro.

[273] Udacity. https://github.com/udacity/self-driving-car-sim.

[274] Dosovitskiy A., Ros G., Codevilla F., Lopez A., Koltun V. (2017). CARLA: An open urban driving simulator. arXiv.

[275] Airsim. https://github.com/Microsoft/AirSim.

[276] Fan H., Zhu F., Liu C., Zhang L., Zhuang L., Li D., Zhu W., Hu J., Li H., Kong Q. (2018). Baidu apollo em motion planner. arXiv.

[277] Deepdrive. https://deepdrive.voyage.auto.

